# A scoping review of studies on secure messaging through patient portals: persistent challenges and potential solutions

**DOI:** 10.1038/s44401-026-00091-2

**Published:** 2026-05-24

**Authors:** Yawen Guo, Di Hu, Yiliang Zhou, Tianchu Lyu, Ziqi Yang, Tera L. Reynolds, Kai Zheng

**Affiliations:** 1 Department of Informatics, University of California, Irvine, CA USA; 2 Department of Information Systems, University of Maryland, Baltimore County, Baltimore, MD USA

**Keywords:** Business and industry, Health care, Medical research, Scientific community, Social sciences

## Abstract

Secure messaging (SM) has seen increasing adoption over the past decade, prompting growing interest in understanding its impact on healthcare delivery. This review examines key themes in the existing SM research, such as usage patterns, perceived benefits, and persistent challenges, to identify research gaps and inform opportunities for sociotechnical solutions in the artificial intelligence (AI) era that could enhance patient–provider communication effectiveness. Searches were conducted in PubMed, Scopus, IEEE Xplore, ACM Digital Library, Cochrane CENTRAL, CINAHL, and Web of Science. Following the PRISMA guideline, we identified 366 relevant peer-reviewed studies published from January 2009 to September 2025. Existing research has primarily investigated (1) the effect of SM use on clinical outcomes, (2) adoption patterns, and (3) user experiences. The literature shows that SM promotes patient engagement, care coordination, and patient–provider communication. However, significant challenges persist, including privacy concerns, limited access for vulnerable populations, patient misuse, and increased clinician burden. Educational initiatives and patient-centered design are essential for promoting appropriate and accessible use of SM. Emerging AI solutions also show promise in enhancing SM use, particularly for message triaging and drafting replies. The integration of such AI solutions must be guided by robust governance frameworks to ensure transparency, maintain trust, and align with evolving clinical, billing, and regulatory environments. Future research should include more diverse care settings and populations, prioritizing the development of equitable sociotechnical tools and interventions that can be seamlessly integrated into clinical workflows.

## Introduction

The Health Information Technology for Economic and Clinical Health (HITECH) Act has promoted the meaningful use of electronic health records (EHRs) to enhance healthcare quality, patient engagement, and data security^1^. Under its Meaningful Use provisions, secure electronic messaging was established as the standard for patient-provider communication and was promoted in support of EHR innovations like telehealth to enhance care between visits^2–4^. Secure messaging (SM), defined as “any electronic communication between a provider and patient that ensures only those parties can access the communication”, is a core feature of patient portals and plays a critical role in the broader landscape of telehealth^5–8^.

SM has become a widely adopted feature in patient portals, supporting patients in self-managing health conditions and coordinating care^9,10^, showing potential to improve patient engagement and provider workflows via enabling multimodal asynchronous information exchange^11^. Despite its growing use, challenges persist in expanding adoption, ensuring effectiveness, and addressing overuse, particularly since the COVID-19 pandemic^12–18^. As SM has taken on greater importance in clinical care, policy responses have followed. For example, CMS introduced billing codes for portal messages that involve medical decision-making and require more than five minutes of clinician time within a seven-day period^11^. These policies acknowledge the clinical value and resource burden of SM while underscoring the need for ongoing evaluation of their impact on care delivery, equity, and provider workload^15,19^.

Machine learning (ML) and deep learning(DL) approaches have long been explored to enhance SM in support of a variety of clinical tasks, including triaging, message classification, and workload prediction^20–22^. More recently, advances in generative artificial intelligence (GenAI) and large language models (LLMs) have prompted EHR integration of these technologies for message drafting and workflow optimization^23–25^, particularly with respect to reduction of provider workload associated with SM^26–29^. Despite considerable enthusiasm, the real-world benefits of such artificial intelligence and machine learning (AIML)-enhanced SM approaches have yet to be firmly established. Such an evaluation presents a two-fold challenge: first, elucidating how SM affects care delivery and clinical workflows, and second, determining the additional impact of AIML on people and these processes.

While several SM studies exist that provide important insights on the former point, many were conducted before the widespread adoption of modern EHRs and digital health technologies and thus reflect an outdated technological landscape. Early reviews examined its potential to enhance patient-provider communication, focusing on user expectations, confidentiality concerns, and technological limitations reflective of the pre-2010 landscape^30–32^. Conversely, more recent reviews highlight specialized aspects rather than a generalized view of the field, covering specific vendor applications^33^, disease- or population-specific implementations^34–36^, or possessing computational or methodological foci^37,38^. A comprehensive understanding of SM’s role in care delivery—its benefits, limitations, and areas of adoption—is essential for determining whether, where, and how AI should be integrated into clinical workflows.

In this article, we therefore aim to systemically understand the current and prospective role of the patient–provider SM communication in clinical care: its benefits, drawbacks, and implications for potential solutions. In doing so, we establish a contemporary foundation to identify opportunities for, and risks inherent to, adoption of AIML-enhanced SM from the perspective of the health system as a whole. We then conclude with further elaboration on several such identified opportunities and risks.

## Results

**Fig. 1:**
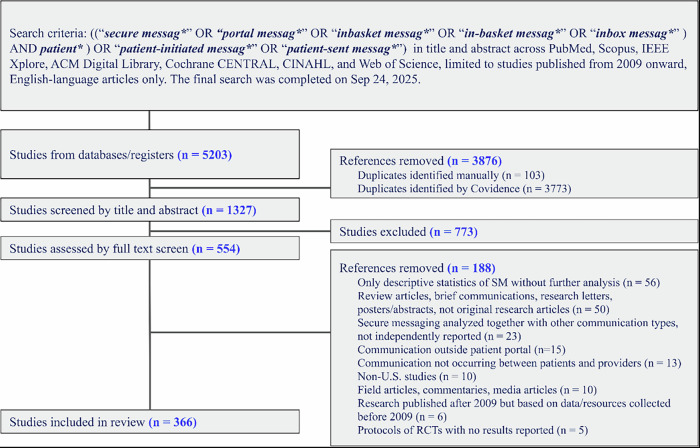
PRISMA-style flow diagram of literature search and study selection. The figure summarizes database searching, duplicate removal, title/abstract screening, full-text eligibility assessment, exclusions, and final inclusion of studies on patient-provider secure messaging.

A search across seven databases yielded 1327 unique studies. A total of 773 studies were removed as they were unrelated to SM after title and abstract screening. After full-text screening of 554 articles, 188 were excluded based on predefined criteria (Fig. 1). We included 366 relevant studies in this review. The process followed PRISMA guidelines to ensure methodological rigor and comprehensive coverage. Agreement between reviewers was substantial at both the title and abstract screening phase (*κ* = 0.67) and the full-text review phase (*κ* = 0.66). All conflicts were resolved through consensus.

**Fig. 2:**
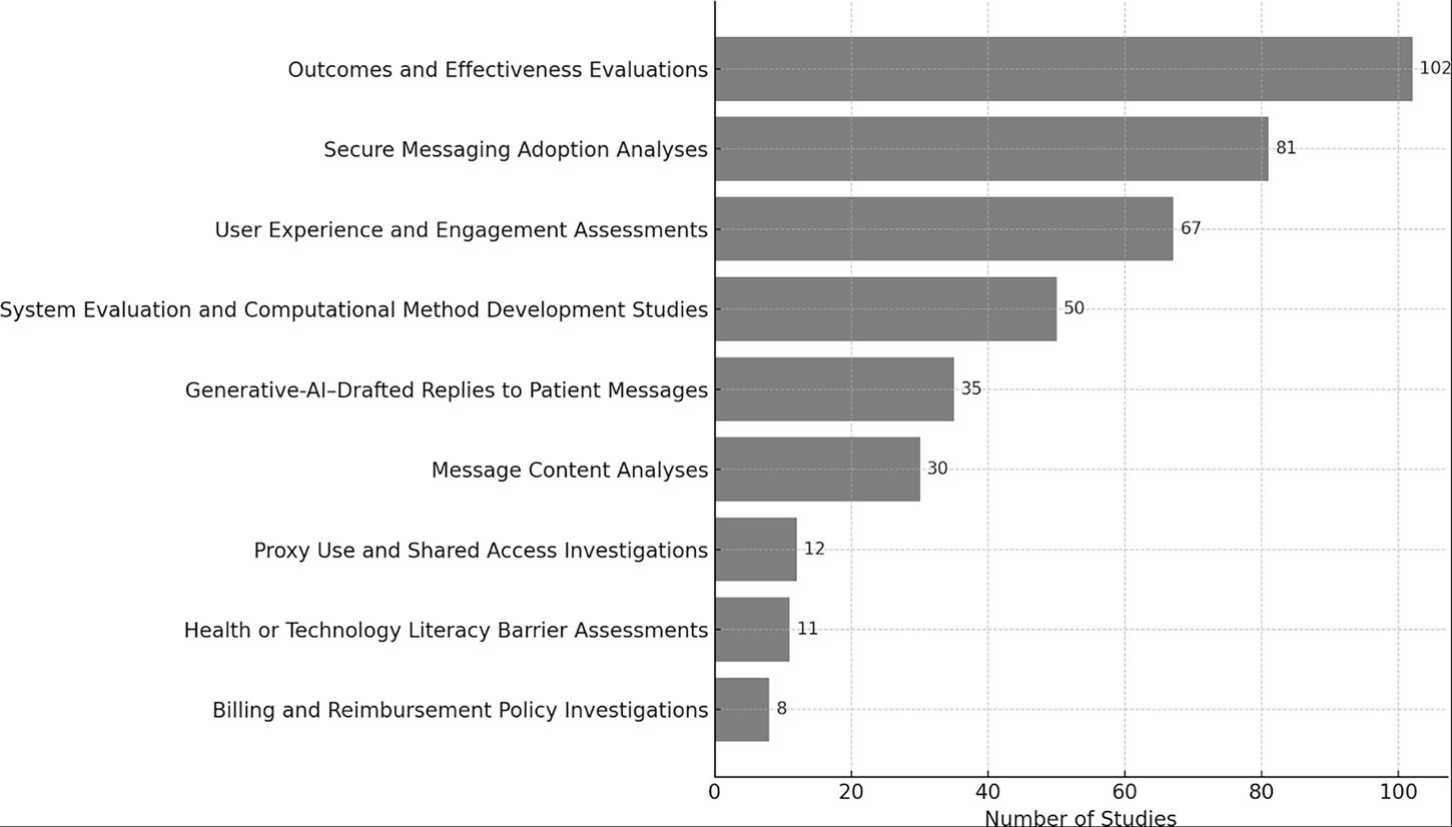
Distribution of Research Themes Across Reviewed Articles (*n* = 366). *Some studies were coded under multiple categories.

Our analysis categorized these 366 studies into nine distinct research themes (Fig. 2), capturing a wide range of topics related to SM within EHRs. The following sections present findings from each category, highlighting key insights on healthcare communication, patient engagement, clinical outcomes, and technological developments.

### Outcomes and effectiveness evaluations (*n* = 102, 27.9%)

Most studies accessing outcomes or effectiveness showed that SM utilization is associated with benefits for patient care and patient, clinician, and researcher work across various clinical conditions, care settings, and patient populations. For example, SM has been found to enhance diabetes care and self-management^39–48^, and improved survival outcomes and symptom monitoring among oncology patients^49,50^. Use of SM has been associated with improved hypertension management via better blood pressure monitoring and medication intensification^51–53^.

Studies assessing the effectiveness of SM outreach and interventions have been overall positive, but somewhat mixed. 12 studies have linked SM with increased rates of vaccination^54–59^, colorectal and hepatitis C screenings^60–62^, pediatric care attendance^58,63,64^, and genetic testing for Alpha-1 Antitrypsin Deficiency^65^. Interventions conducted via SM also showed potential association with improved medication adherence among patients, including for smoking cessation^61–65^, chronic myeloid leukemia^66^, spina bifida^67^, and depression^68^. In surgical contexts, simplified dismissal instructions after prostatectomy were reported to be associated with fewer postoperative patient-initiated messages and ED visits^69^. Among elderly patients, SM communication has been found to facilitate care planning and advance directives completion^70^. In pediatric young adult settings, SM has been reported to support medical decision-making^71^, deliver educational interventions in suicide prevention and HIV care^72–76^, and facilitate follow-ups following office-based circumcision^77,78^.

Secure messaging related usage metrics has been used as an indicator of hospitalist efficiency and patient portal engagement^79–83^. It has been reported to influence clinical workflow by increasing patient engagement and supporting the adoption of e-visits^75,84–90^. However, a higher volume of SM interactions was reported to reduce new patient intake capacity^84,91^. While its association with emergency visits varies by conditions^92–94^, SM has generally been linked with more efficient primary care workflows, including lower urgent care utilization and fewer unnecessary visits^95–99^. Studies have found that provider burnout remains a concern because of large message volume^100^, while message characteristics, such as sentiment scores, do not appear to be a major contributing factor^101^. SM has also demonstrated effectiveness as a clinical research recruitment tool, achieving high response rates compared to mail, phone, and social media methods across diverse clinical trials^102–111^.

Studies comparing SM with traditional communication methods highlight varied user preferences influenced by clinical context and demographics, suggesting that tailored communication approaches are essential^108,112–123^. Studies report lower response rates of SM for COVID-19 research recruitment and preventive screening when compared to traditional in-person, phone, or mail communication^112,113,124,125^. Influenza vaccination studies show mixed results, with some reporting associations with higher uptake^116,126,127^, while others find no population-level benefits from SM reminders^128,129^ or content-tailored SM^130^. Although certain studies have suggested benefits of SM interventions, others report no significant advantage of SM outreach for smoking cessation^74,131,132^ or HIV medication refills^133,134^. Similarly, a randomized trial found that a single SM sent from a provider to a patient was not associated with an increase in HbA1c follow-up testing^135^.

### Secure messaging adoption analyses (*n* = 81, 22.1%)

Secure messaging adoption has been expanding substantially since its introduction around 2010 and is now reported as the most used patient portal function^136–139^. During this time, most office-based physicians adopted SM, although uptake was lower among solo and non-primary care providers^140–142^. Disparities emerged following federal incentives, with certified health IT use positively associated with SM adoption, as well as providers’ expectations that SM would improve performance and their overall attitudes toward its use^143–145^. Research shows SM volume continues to rise but per capita rates have plateaued, indicating SM adoption is still expanding^142,146–152^. The COVID-19 pandemic triggered a major surge in use, and persistent high volume has been suggested to contribute to multitasking, workflow disruptions, and potential medical errors^153–162^. A national study found that only 1.9% of veterans used SM during early adoption, with uptake varying by condition and demographics^163^.

Patient SM usage patterns vary across demographics. Patient users are more often younger^88,164–172^, women^165,168,170,173–177^, Caucasian^88,165,167,168,174,176,178,179^, married^88,106,167,168,176,180^, employed^88^, privately insured^88,144,170,181,182^, and residing in high socioeconomic neighborhoods^88,143,166,183^ with higher education levels^164,168,184,185^. Many also have poor physical^164^ or mental health conditions^174,186,187^, and higher levels of morbidity or comorbidity^166–168,170,187,188^. Active patient users are generally English speaking^176,179,189^, with high EHR usage and more outpatient visits^190–193^. Portal refresh behaviors also shape adoption patterns, with patients who more frequently refreshed test results more often initiating SM^194^. Among adolescent patients, SM users were more often female or transgender with prior portal experience^195^. In contrast, lower SM engagement has been observed among underrepresented minorities^139,144,196–198^, veterans experiencing homelessness^186,199,200^, individuals with limited English proficiency or low health literacy^188,201–204^, and patients facing poor internet access and other digital disparities^166,174,184,205,206^.

Patient–provider interactions shape SM adoption, and have been described as being influenced by behavioral health models, clinical roles, and the characteristics of both patients and providers^207,208^. Female patients have been reported to be more likely to request medical guidance via SM but receive fewer confirmations in response^209^. Female physicians are more often than male physicians to send and receive SM, which may in turn contribute to higher rates of burnout among female providers^173,210–213^. Adoption patterns also vary by clinical specialty, with some studies reporting higher message exchange volumes in obstetrics and gynecology, dermatology, and medicine^142^. However, among pediatric surgical specialists, a recent analysis found no significant differences in inbox message volume or message length after adjusting for case complexity, suggesting that gender disparities in messaging may be context-dependent and vary by specialty^214^.

### User experience and engagement assessments (*n* = 67, 18.3%)

Studies evaluating user experiences report high satisfaction among patients^215–219^, and strong enthusiasm from providers^220,221^, particularly in relation to medication refills, appointment scheduling, test result access, clinical inquiries^14,222–229^. Patients view SM as a tool that complements face-to-face communication, especially regarding sensitive issues like sexually transmitted diseases(STDs) and erectile dysfunction^215,230,231^. SM use has also been reported to improve perceived access to care^14,157,222,230,232–234^ and to strengthen patient-provider relationships^235^. Providers have described valuing SM for its potential role in improving workflow, care coordination, and enabling remote work^232,236^.

However, patients have raised concerns about provider burden from uncompensated SM interactions, delayed responses, unclear messages, and difficulty reaching specific providers–issues that may contribute to tracking challenges and inconsistent communication^12,225,237–243^. Providers have likewise reported increased workload due to time-intensive message management^234,241,242,244–251^, and stress from inappropriate or emotionally challenging messages^12–14,225,252^. Findings from several studies suggest that message-related work created additional burdens, such as constant redirection of attention, lengthy inbox management, and heavier mental workload—factors that have been associated with reduced efficiency and responsiveness^244,247,253–256^. A recent study of over 1700 physicians reported that patient messages requesting medical advice were associated with significantly increased after-hours work, with specialists experiencing a greater burden than primary care physicians^257^. Additional concerns noted in the literature include liability risks^246^, limited effectiveness of SM in inpatient settings^258^, and inadequate training among medical trainees in using SM^259^.

User-perceived barriers to SM adoption include patient misconceptions and previous negative experiences—such as uncertainty about what counts as a ‘non-urgent’ message or concerns about whether physicians have sufficient time to devote to messaging^140,230,260^. Patients have also reported challenges related to message ambiguity and complexity, particularly among elderly patients with lower literacy^140,236,261^. Other barriers described in the literature from the provider perspective include delegation of messaging tasks that complicate communication^13^, limited technology and internet access^13,205,224^, and unclear guidelines for messaging use^140,262,263^. Further work has similarly characterized inbox tasks as fragmented and labor-intensive, often requiring additional articulation work to clarify conflicting or redundant requests^264^. One study reported that the use of medical scribes was not associated with reductions in time to completion of SM^265^. Proposed solutions emphasize education and systematic training to instill more efficient inbox management habits of providers^233,239,253,266–270^, accessible user interfaces with clear messaging instructions^140,255,262^, and AI-driven tools designed to improve patient literacy and support physician workflows^140,271,272^. Additional recommendations include strategic message management practices such as team-based message sharing^225,233,241,253,273^, crafting clear and empathetic messages^274,275^, and standardizing administrative processes to enhance efficiency and reduce clinician burden^267^.

### System evaluation and computational method development studies (*n* = 50, 13.7%)

Early evaluations of SM systems reported rapid consultations turnaround times during pilot e-visit deployments^276^. However, later studies identified persistent system-level usability and access challenges including delayed message response times, suggesting limitations in platform responsiveness and interface design^277,278^. System analyses using signal and log data highlighted rising message volumes and uneven clinician workload distribution, with primary care physicians and nurse practitioners handling most communication during standard work hours^169,279^. Usability research in hospital settings identified functionality gaps, such as clinical document sharing errors^280^, while broader evaluations linked increased SM use to patient access to EHR data^281^.

Recent studies leverage patient–provider SM data to improve clinical efficiency and communication using computational methods. Theory-informed tools—designed to enhance digital engagement, support content creation, and implement feedback mechanisms—were associated with increased portal use among users with diverse literacy and numeracy skills^275,282–284^. Automated assessments of readability and health literacy have provided insights into message complexity, informing both clinical care and population-level interventions^285–289^. Framework-based approaches include SPICE, which emphasizes support, partnership, and information-giving, and a Chain-of-Thought routing framework leveraging LLMs; both illustrate structured strategies intended to improve message handling and patient-centered communication^233,290^. An extended Technology Acceptance Model that included affordances and communication efficacy found that young adults’ intentions to use portal messaging were positively associated with editability and communication efficacy, but negatively associated with persistence^291^.

Rule-based, natural language processing(NLP), deep learning(DL) models have been developed to triage SM by risk and urgency^22^, classify information type (e.g., clinical, medical, logistical, or social)^20,21^, detect medical decision complexity^26–29^, and identify caregivers and caregiving network among people living with dementia^292^. A prospective deployment of a fine-tuned NLP classifier for automated routing reported reduction in response times and staff interactions while maintaining high accuracy across message categories^293^. They were also applied to identify primary patient concerns, social needs, and mental health crises^294–304^. Efforts to extract actionable insights from messages include building FHIR(Fast Healthcare Interoperability Resources)-aligned data models to standardize SM content and enable downstream NLP analyses, integrated semantic and contextual features for improved categorization, and combined message-based features with predictive models to support clinical decision-making^305–308^. Additionally, vector-based representation techniques have been explored with a focus on optimizing word embeddings for small, domain-specific datasets^309^, and prompt engineering has been used to generate synthetic drug-related messages to reveal language patterns^310^. More recently, studies have applied LLMs to tasks such as triaging patient messages to appropriate clinics or urgency levels, detecting knowledge questions, tailoring patient support tools for chronic disease management, and identifying emergencies in real time^311–315^.

### Generative-AI–drafted replies to patient messages (*n* = 35, 9.6%)

A rapidly growing body of 35 studies published between 2023 and 2025 has examined the use of large language models (LLMs) to generate draft responses to patient portal messages. Live implementations in EHR pilots occurred mainly in Epic in-basket workflows, often pairing GPT-4 with institutional customization^23,299,316–320^, while the majority of studies used simulated or de-identified message sets across diverse specialties^24,25,321–337^.

AI-generated replies were often rated as comparable to, or in some cases better than, clinician-authored responses in terms of informational adequacy and clarity, and several studies highlighted enhanced empathy, particularly in GPT-4 outputs^25,317,322,328–331,338–340^. Model benchmarking further reported GPT-4’s consistent outperformance of earlier LLMs across domains^321,326^. Evidence from real-world pilots showed low adoption of draft functions and near-universal need for clinician edits before sending. Drafted messages were generally longer than clinician-authored replies, and objective time savings were inconsistent—though many clinicians perceived reduced burden^23,316–318,341^. Metrics such as edit distance and message length were commonly used to quantify these patterns^337^. Several studies emphasized that iterative prompt engineering and contextual grounding—for example, incorporating the most recent assessment and plan—were associated with improvements in draft quality and clinician acceptance^316,326,332,341,342^. Human-in-the-loop prompt refinement and structured prompt templates were also reported as effective strategies^319,333^.

Clinicians described drafts as useful starting points that could reduce cognitive load, though some expressed concerns about over-reliance^255,323,328,330^. Patients in blinded evaluations frequently favored AI replies for empathy and readability^317,325,328^, while mixed feedback emerged around the tone of lengthy or formal responses^322,324^. Risks were documented across multiple contexts, including hallucinations, incoherence, and misinterpretation of clinical nuance^25,321,324,331,334,343^. These findings underscore the necessity of clinician oversight in all implementations^329^. Participants across several studies were often unable to reliably distinguish AI-authored from human-authored replies^325,328^. While disclosure of AI involvement slightly reduced patient satisfaction, transparency was widely viewed as necessary for trust and accountability^323,332,344,345^.

### Message content analyses (*n* = 30, 8.2%)

Studies analyzing SM content have used both qualitative and computational methods to examine communication patterns. Common topics include medication-related issues, appointment scheduling, lab results, referrals, and other administrative tasks^39,114,155,165,346–355^. Patients have been observed to use SM to report symptoms, express psychosocial concerns, seek medical advice, and convey appreciation or complaints^114,155,165,175,349,350,352,356–361^. SM content also reveals communication around sensitive health topics such as STDs and cannabis use^215,362^. In surgical populations, SM content has focused on drains, wounds, pain, and activity restrictions, with message patterns varying by patient age and mental health status^363^. Tone analysis reveals that most messages are neutral and respectful, though cyber-incivility and problematic language occasionally challenge professional standards^252,364,365^. Evidence suggests that provider replies may include less language that fosters partnership or emotional support^366^, and provider-initiated messages often reflect greater clinical complexity^367^. Recent work has also applied large-scale NLP to patient portal messages to surface patient-driven research priorities in cancer care, with approximately one-third of the AI-generated topics rated by clinicians as highly meaningful and novel^368^.

### Proxy use and shared access investigations (*n* = 12, 3.3%)

This body of research focuses on patient portal use among adolescents with their guardians, and elderly patients with their caregivers. Studies have highlighted benefits such as convenient communication and patient empowerment, but also raised concerns about privacy and confidentiality—particularly for adolescents under the Cures Act Final Rule^369,370^. Analyses indicate that more than half of messages from adolescent accounts are accessed by guardians, underscoring privacy management challenges^371,372^. For elderly populations, research has consistently reported caregiver use of portals and SM as proxies, reflecting significant reliance on caregiver support^355,373–375^. Caregiver proxies have been observed to more commonly manage portals for older adults, males, non-white patients, non-English speakers, noncitizens, and those with public insurance^54^. To address these challenges, recent studies developed computational methods to identify non-patient authors, aimed at improving management efficiency and strengthening privacy safeguards in patient portal communications^54,188,376^.

### Health or technology literacy barrier assessments (*n* = 11, 3.0%)

Several studies have emphasized the importance of health and digital literacy for the adoption and effective use of SM. Research indicates that while patients often write in simpler language, a mismatch in communication complexity has been observed in a notable portion of exchanges^285^. Studies have also identified disparities linked to the digital divide, highlighting barriers such as low digital literacy^139,202^. Providers have reported technology-related difficulties, including complex login procedures and frequent log-offs^12^. Investigations into SM complexity suggest that automated feedback mechanisms may support message clarity and user education, thereby helping providers communicate more effectively^275^. Computational models using linguistic analysis of patient messages have also shown promise in assessing health literacy and identifying educational needs automatically^236,275,286–289,377^.

### Billing and reimbursement policy investigations (*n* = 8, 2.2%)

Recent studies examining billing policies for SM e-visits have highlighted changes in clinician billing behaviors and message usage patterns following the 2020 decision by CMS to allow billing for SM. Initial assessments reported modest declines in message volume after CMS’s decision and low physician participation in billing activities^15,19,378–380^, even though providers generally accepted the new model^380^. Emerging research has proposed refining billing strategies using predictive models based on clinical complexity rather than time spent, with the aim of improving billing accuracy and effectiveness^29,381^. Investigations in specialty areas, such as ophthalmology, have reported a temporary increase in billing during the pandemic, underscoring challenges in sustaining equitable SM billing across diverse healthcare contexts^382^.

## Discussion

This scoping review systematically maps the evolving landscape of SM research, emphasizing its role in patient–provider communication and the increasing influence of AI in shaping its implementation. SM has shown clear benefits in enhancing access, continuity, and care coordination. However, challenges around equity, usability, and provider burden persist, underscoring the need for coordinated efforts among all key stakeholders, including patients, providers, researchers, and policymakers. To guide future work, we discuss findings across four major domains, reflecting the research themes identified in our results.

Across the included studies, the majority has been conducted within single healthcare institutions, particularly emphasizing primary care settings. Investigations often target specific populations: e.g., older adults and veterans^100,243,355,373^, or patients with specific diseases, such as chronic illnesses^54,238,352^. Concurrently, there has been limited exploration of SM’s benefits and limitations beyond primary care, such as in emergency or specialty settings. While the bulk of SM’s applicability is in primary care^278,285,301,344^, they may also be useful in specialty settings^94,307^. These investigations on SM systems in limited, focused, settings inherently also limit the broader applicability and generalizability of findings. Future research should therefore diversify study populations, broaden the range of health conditions examined, and include varied care settings.

Although SM offers important opportunities to strengthen patient–provider communication^30,31,34^, many patient-side barriers persist, including misconceptions about appropriate use, inequities in adoption, and privacy concerns.

Patients often hold misconceptions about SM, influenced by negative experiences with telehealth, uncertainty about who receives their messages, and concerns over privacy and system responsiveness^140,230^. Some patients hesitate to use SM due to fear of overburdening their providers, while others misuse it for urgent needs^238,240^. Although computational methods such as urgency classification have been developed to support triage^349^, they alone are insufficient to ensure appropriate use. AI-based tools have the potential to guide patients in real time by suggesting appropriate message categories, clarifying urgency, or simplifying language. However, these solutions must be paired with user education to be effective, as patients need guidance on when and how to use SM appropriately^12,225,239,242^. Some health systems have begun embedding brief guidance within patient portals or visit summaries, yet few efforts are tailored to individuals with limited digital literacy, language barriers, or disabilities. Furthermore, little is known about the effectiveness of these approaches or whether they reach the patients most in need. Clear communication at the point of care, e.g., setting expectations about appropriate message use during visits, may help address these misunderstandings without adding new technological burden. Finally, additional safeguards must be in place to protect patient privacy when messages are accessed or sent by non-patient senders, such as caregivers, guardians of adolescents, or individuals assisting patients with disabilities^355,370,371,383^. Potential strategies include role-based access controls, clear proxy account policies, and automated alerts to help patients maintain awareness of who can view or send messages on their behalf.

Non-English speakers and the elderly continue to face technical barriers that limit their effective use of SM^54,188^. Addressing these challenges requires patient-centered design that incorporates user-friendly interfaces and supports low-literacy communication to enhance functionality and accessibility for diverse populations^364,366,384–387^. Individuals from low socioeconomic backgrounds often face barriers to accessing SM, particularly when care is delivered across multiple providers^88,143,166^. Current interventions are often passive, relying on patient-initiated engagement, which may inadvertently exclude underrepresented groups who are unaware of or hesitant to use SM. While some outreach efforts exist, such as reminders for vaccinations or preventive screenings, few are tailored to the needs of specific populations. Future directions include co-designed educational materials, multilingual and plain-language prompts, and accessibility features, alongside rigorous evaluation of their impact. AI-powered tools may further support equity by delivering simplified language, visual aids, and personalized educational content to improve comprehension and engagement.

Beyond patient-facing barriers, our review identified substantial strain on providers, driven by increasing message volumes, unclear workflows, and limited reimbursement mechanisms.

Misuse of SM for urgent issues can shift liability and clinical responsibility to providers, potentially compromising patient care^252,365^. Patients’ use of inappropriate language or emotionally charged messages may contribute to a stressful professional environment and emotional exhaustion^252,365^. Beyond emotional strain, operational burdens include frequent attention shifts, prolonged inbox management, and increased cognitive load, which can reduce the quality and timeliness of responses^244,247,253^. The surge in message volume further intensified these pressures, often extending work into personal time and increasing physician burnout^225,241,256,388,389^. Disparities in SM use between physician- and hospital-owned practices suggest that organizational structures also shape provider engagement^178^. To address these challenges, some interventions have introduced structured workflows or support systems, such as team-based inbox management or message triage protocols, but few studies have systematically evaluated their effectiveness in reducing workload or improving care delivery. There is also a role for provider-facing education to ensure consistent messaging around SM use during clinical encounters^233,239,253,266–270^. Future research should assess the comparative effectiveness of these workflow interventions and identify conditions under which AI-driven support, such as automated triage or message summarization, can reduce workload without undermining clinical judgment. Organizational strategies such as clearer role delineation and protected time for messaging are critical components of reducing provider burden. However, AI-generated drafts may introduce new forms of cognitive and time burden, underscoring the importance of human-centered workflow design to ensure net workload reduction^390^. A balanced approach that integrates automation with clinician-centered workflow design is needed to alleviate provider burden.

While early billing policies for SM focused on clinician time and decision-making complexity, ongoing implementation has raised broader concerns around equity, resource allocation, and communication dynamics^15,19^. Modest physician engagement and reduced patient messaging suggest hesitancy toward new billing protocols and potential shifts in patient behavior^378,379^. Predictive modeling based on clinical complexity has offered strategies to align care delivery with billing practices^381^. Although AI-enhanced billing tools could theoretically analyze message content and estimate clinical effort, they remain largely untested^391^. As these technologies evolve, it is important to monitor their impact on provider documentation and patient access. Future research should further evaluate how providers and patients adapt to SM billing and explore how AI can contribute to transparent and sustainable reimbursement models.

Computational and AI-driven approaches are increasingly being explored to manage message volume and workflow, improve literacy support, and generate draft replies.

For current computationally augmented SM implementations, the primary focus has been on automated triage and response prioritization. Studies have applied approaches ranging from traditional ML models such as logistic regression, support vector machines, and random forests^21,22,26^ to DL architectures, particularly BERT and its variants^298,392^. These methods have been used to classify message urgency and decision complexity^300,376,393^, and to assess readability or literacy mismatches in patient–provider communication^286–288^. While most work has been limited to retrospective analyses or small-scale pilots, findings suggest promising utility for identifying high-risk messages, enhancing literacy support, and improving workflow efficiency. Future directions include adapting these tools for live clinical workflows, integrating them with structured EHR data, and examining their impact on equity (e.g., whether automated triage inadvertently deprioritizes messages from underserved populations). As LLM-based methods are now being applied to similar SM tasks^311–315^, future research should prioritize rigorous evaluation of their accuracy and safety, and determine how they can complement existing ML and DL approaches^37^.

The integration of GenAI and LLMs into SM offers new opportunities to improve efficiency and reduce clinician burden, especially in the management of in-basket messages^386,394^. While providers often prefer human-generated messages for their readability and empathy^25,317,337^, patients are generally receptive to GenAI-generated messages, especially when tone and context are appropriately managed^327^. Recent research has begun exploring GenAI’s feasibility across varied message types and clinical scenarios to improve applicability and trust in message drafting^323,329,341,342^. Pilot studies are also testing GenAI for triage, response generation, and workload redistribution within SM systems^23,316^. However, GenAI outputs can include hallucinations or confabulations, such as incorrect clinical facts or inappropriate recommendations, which may introduce patient safety risks without robust human oversight and verification^395^. In addition, these tools may be misused by generating responses beyond a clinician’s scope, over-automating sensitive communication, or mishandling urgent symptoms^396^. While early studies have explored GenAI feasibility, widespread integration will require more robust evaluation of message quality and broader impacts on communication safety, patient–provider relationships, and workflow efficiency. As SM continues to evolve, human-centered governance will be essential to ensure that GenAI enhances care while maintaining trust and accountability.

This review included only full-length archival articles on empirical studies, excluding brief reports, research letters, and industry case studies that may have provided early insights into emerging trends. Future reviews should consider these formats to broaden analytical scope. Our search approach focused on SM terminology common in portal-based systems, which may have excluded earlier studies using different descriptors such as “electronic messages”. Our synthesis may be biased toward more favorable conclusions about concurrent SM effectiveness, because early implementations often faced lower adoption and less integrated workflows. As a result, the benefits summarized here may represent an upper-bound estimate relative to the full historical evidence base. We also limited inclusion to patient–provider communication via portals, excluding studies on stand-alone SM applications and interprofessional communication (e.g., physician–nurse exchanges) to maintain a clear focus. Future research should examine SM in alternative platforms and team-based care contexts to better capture its broader impact on healthcare communication and coordination.

This scoping review synthesized literature on patient–provider secure messaging (SM) within patient portal, highlighting key themes including user perspectives, communication patterns, clinical outcomes, and technology adoption. While SM offers clear benefits for patient engagement and care coordination, challenges persist around provider workload, digital access, and equitable implementation. AI-assisted tools show promise in addressing these barriers but must be paired with thorough evaluation and careful integration into clinical workflows. We underscore the need for user-centered system design, targeted education to improve digital and health literacy, and informed policy development regarding billing and physician burden. Future research should further explore how LLMs and GenAI can be responsibly applied to support secure and efficient patient–provider communication.

## Methods

### Literature search and selection

We conducted a literature search across PubMed, Scopus, IEEE Xplore, ACM Digital Library, Cochrane CENTRAL, CINAHL, and Web of Science. The final search strategy combined the terms (“secure messag*” OR “portal messag*” OR “inbasket messag*” OR “in-basket messag*” OR “inbox messag*” OR “patient-initiated messag*” OR “patient-sent messag*”) AND patient* in the title and abstract, restricted to studies written in English and published from 2009 onwards. A detailed breakdown of search strategies for each database, including syntax variations and filters, is provided in the Supplementary Table 1. The search process was conducted in four waves to ensure comprehensive coverage of the literature over the study period. The first search was completed in December 2023 and provided the initial corpus used to pilot and refine the inclusion and exclusion criteria. A second and third search were conducted in October 2024 and April 2025 to update the corpus and incorporate new publications that had appeared in the interim, while also allowing refinement of coding practices and terminology. The final search was completed in September 2025, ensuring that the review reflects the most recent body of evidence. Results in this manuscript are based on the combined set of studies identified across all four searches. The titles and abstracts of the identified articles were independently screened by YG and another author (DH, YZ, TL, or ZY). Articles identified as relevant after the title and abstract screening process then underwent a full-text review to determine final inclusion(YG and DH). Any disagreements were resolved through consensus meetings with a third author (KZ). Inter-rater reliability was assessed using Cohen’s κ in Covidence for both the title and abstract screening stage and the full-text review stage. Because all records from different search waves were uploaded together, the κ values represent overall agreement across the full screening process.

We included original, peer-reviewed full-length empirical studies in which patient–provider SM was a central focus. We limited inclusion to U.S.-based studies to improve comparability, because portal-based secure messaging is shaped by U.S.-specific EHR adoption, privacy, and reimbursement contexts. We excluded studies that: (i) did not examine SM directly, (ii) analyzed communication solely between providers, (iii) combined SM with other modalities (e.g., telephone or email) without separate reporting, (iv) were conducted outside the U.S., or (v) reported only basic descriptive metrics of SM use (e.g., counts or enrollment) without analytical depth. For example, studies reporting only annual message volumes without linking them to outcomes, content, or user experience were excluded.

### Data extraction

To formulate the codebook, YG and DH iteratively coded sets of 10 articles, followed by consensus discussions with KZ to refine codes. The nine final categories presented in Table 1 were derived inductively from recurring themes that emerged during the review of eligible studies. Once initial themes were identified, all authors, who actively conduct informatics research on clinical informatics, reviewed the categories and reached consensus on their definitions and boundaries. These nine categories— *Outcomes and Effectiveness Evaluations, Secure Messaging Adoption Analyses, User Experience and Engagement Assessments, Message Content Analyses, System Evaluation and Computational Method Development Studies, Generative-AI–Drafted Replies to Patient Messages, Proxy Use and Shared Access Investigations, Health and Technology Literacy Barrier Assessments*, and *Billing and Reimbursement Policy Investigations*—formed the framework for categorizing research themes and synthesizing findings.

**Table 1:** Coding Framework for SM research themes

Code category	Example codes
Outcomes and Effectiveness Evaluations	use of SM as an intervention; SM as a predictive tool; evaluation of clinical or behavioral outcomes
Secure Messaging Adoption Analyses	SM adoption rates; proportion of SM users among EHR enrollees; usage behavior analysis
User Experience and Engagement Assessments	user perspective/satisfaction/preferences; comparative analysis of SM vs. other methods; strategies to improve engagement
System Evaluation and Computational Method Development Studies	development of SM-related computational tools; evaluation of SM system functionality; user interface design
Generative-AI–Drafted Replies to Patient Messages	evaluation and user perceptions of AI vs clinician response quality; assessment of utilization; prompt engineering and context grounding; disclosure of AI involvement and authorship recognition; identification of risks and safety concerns
Message Content Analyses	sentiment analysis; topic or theme analysis; linguistic analysis
Proxy Use and Shared Access Investigations	examination of proxy message authorship; issues related to shared access to SM
Health and Technology Literacy Barrier Assessments	analysis of patient health literacy and education; technological literacy and associated barriers
Billing and Reimbursement Policy Investigations	evaluation of e-visit billing codes; reimbursement-related SM usage trends; financial incentives and barriers

Two of the three researchers (YG, DH, and YZ) independently coded each article, and discrepancies were resolved through consensus or input from the third reviewer (KZ). A structured coding scheme was developed with open-text fields to capture additional details. We coded research themes using a multi-select field comprising nine inductively derived categories, with the study’s objective recorded verbatim and an “other” option for edge cases. When studies spanned multiple thematic areas, we assigned multiple themes based on the paper’s objectives and the primary outcomes described in the *Methods* and *Results*. For example, papers reporting both clinical outcomes and message utilization metrics were coded under *Outcomes and Effectiveness Evaluations* and *Secure Messaging Adoption Analyses*, guided by the Results endpoints. Papers describing development of automated triage tools plus evaluation of user experience were coded under *System Evaluation and Computational Method Development Studies* and *User Experience and Engagement Assessments*, based on the Methods and Results content. In practice, although this manuscript reports primarily on thematic findings due to the large scale and heterogeneity of included studies, the extraction instrument also captured study design, data scope, data source, and site scope (single-site vs. multi-site), as well as population, clinical domain, and healthcare setting. Outcomes, key results, and author-stated recommendations were recorded in structured free-text fields, with the full instrument available in Supplementary Table 2.

## Supplementary information

Supplementary information

## Data Availability

The data analyzed in this study were derived exclusively from previously published literature. The extracted and synthesized data supporting the findings of this review are available from the corresponding author upon reasonable request.

## References

[1] Rights (OCR) O for C. HITECH Act Enforcement Interim Final Rule 2009. https://www.hhs.gov/hipaa/for-professionals/special-topics/hitech-act-enforcement-interim-final-rule/index.html (accessed 5 November 2024).

[2] The Federal Government Has Put Billions into Promoting Electronic Health Record Use: How Is It Going? n.d. https://www.commonwealthfund.org/publications/newsletter-article/federal-government-has-put-billions-promoting-electronic-health (accessed 5 November 2024).

[3] What Are Electronic Health Records (EHRs)? | HealthIT.gov n.d. https://www.healthit.gov/topic/health-it-and-health-information-exchange-basics/what-are-electronic-health-records-ehrs (accessed 5 November 2024).

[4] Blumenthal, D. & Tavenner, M. The “meaningful use” regulation for electronic health records. *N. Engl. J. Med.* **363**, 501–504, 10.1056/NEJMp1006114 (2010). 20647183 10.1056/NEJMp1006114

[5] Konttila, J. et al. Healthcare professionals’ competence in digitalisation: a systematic review. *J. Clin. Nurs.* **28**, 745–761, 10.1111/jocn.14710 (2019). 30376199 10.1111/jocn.14710

[6] Yeung, A. W. K. et al. The promise of digital healthcare technologies. *Front. Public Health* **11**. 10.3389/fpubh.2023.1196596. (2023). 10.3389/fpubh.2023.1196596 PMC10562722 37822534

[7] Tuckson, R. V., Edmunds, M. & Hodgkins, M. L. Telehealth. *N. Engl. J. Med.* **377**, 1585–1592, 10.1056/NEJMsr1503323 (2017). 29045204 10.1056/NEJMsr1503323

[8] Eligible Professional’s Guide to STAGE 2 of the EHR Incentive Programs n.d.

[9] Say Goodbye to Paper and Pagers: Modernizing Clinical Communication | Epic n.d. https://www.epic.com/epic/post/say-goodbye-to-paper-and-pagers-modernizing-clinical-communication-with-secure-chat/ (accessed 5 November 2024).

[10] Secure Messaging Component n.d. https://cernprphelp.cernerworks.com/Help/default.aspx?pg=11 (accessed 5 November 2024).

[11] MEDICARE TELEMEDICINE HEALTH CARE PROVIDER FACT SHEET | CMS n.d. https://www.cms.gov/newsroom/fact-sheets/medicare-telemedicine-health-care-provider-fact-sheet (accessed 5 November 2024).

[12] Ozkaynak, M. et al. Examining the multi-level fit between work and technology in a secure messaging implementation. *AMIA Annu. Symp. Proc.* **2014**, 954–962 (2014). 25954403 PMC4419966

[13] Haun, J. N. et al. Using electronic data collection platforms to assess complementary and integrative health patient-reported outcomes: feasibility project. *JMIR Med. Inf.* **8**, e15609, 10.2196/15609 (2020). 10.2196/15609 PMC7381258 32589163

[14] Sisk, B. A. et al. Oncology clinicians’ perspectives on online patient portal use in pediatric and adolescent cancer. *JCO Clin. Cancer Inf.* **7**, e2300124, 10.1200/CCI.23.00124 (2023). 10.1200/CCI.23.00124 37972324

[15] Liu, T. et al. National trends in billing patient portal messages as e-visit services in traditional Medicare. *Health Aff. Sch.* **2**, qxae040, 10.1093/haschl/qxae040 (2024). 38756169 10.1093/haschl/qxae040 PMC11034524

[16] Soekijad, M. Efficiency paradox: introducing secure messaging in outpatient care. *Soc. Sci. Med.* **365**. 10.1016/j.socscimed.2024.117578. (2025). 10.1016/j.socscimed.2024.117578 39642582

[17] Fogg, J.F. & Sinsky, C.A. In-basket reduction: a multiyear pragmatic approach to lessen the work burden of primary care physicians. *NEJM Catal.* **4**. 10.1056/CAT.22.0438. (2023)

[18] Kurek, A. et al. The “Inboxologist” — A novel approach to in-basket management in primary care. *NEJM Catal.* **5**. 10.1056/CAT.24.0133. .(2024)

[19] Holmgren, A.J. et al. Changes in secure messaging after implementation of billing E-visits by demographic group. *JAMA Netw Open* **7**, e2427053. 10.1001/jamanetworkopen.2024.27053. (2024). 10.1001/jamanetworkopen.2024.27053 PMC11316232 39120906

[20] Cronin, R. M. et al. Automated classification of consumer health information needs in patient portal messages. *AMIA Annu. Symp. Proc.* **2015**, 1861–1870 (2015). 26958285 PMC4765690

[21] Cronin, R. M. et al. A comparison of rule-based and machine learning approaches for classifying patient portal messages. *Int. J. Med. Inf.* **105**, 110–120, 10.1016/j.ijmedinf.2017.06.004 (2017). 10.1016/j.ijmedinf.2017.06.004 PMC5546247 28750904

[22] Chen, J. et al. Detecting hypoglycemia incidents reported in patients’ secure messages: using cost-sensitive learning and oversampling to reduce data imbalance. *J. Med. Internet Res.* **21**, e11990, 10.2196/11990 (2019). 30855231 10.2196/11990 PMC6431826

[23] Tai-Seale, M. et al. AI-Generated draft replies integrated into health records and physicians’ electronic communication. *JAMA Netw. Open* **7**, e246565, 10.1001/jamanetworkopen.2024.6565 (2024). 38619840 10.1001/jamanetworkopen.2024.6565 PMC11019394

[24] Afshar, M. et al. Prompt engineering with a large language model to assist providers in responding to patient inquiries: a real-time implementation in the electronic health record. *JAMIA Open* **7**, ooae080, 10.1093/jamiaopen/ooae080 (2024). 39166170 10.1093/jamiaopen/ooae080 PMC11335368

[25] Baxter, S. L. et al. Generative artificial intelligence responses to patient messages in the electronic health record: early lessons learned. *JAMIA Open* **7**, ooae028, 10.1093/jamiaopen/ooae028 (2024). 38601475 10.1093/jamiaopen/ooae028 PMC11006101

[26] Sulieman, L., Robinson, J. R. & Jackson, G. P. Automating the classification of complexity of medical decision-making in patient-provider messaging in a patient portal. *J. Surg. Res.* **255**, 224–232, 10.1016/j.jss.2020.05.039 (2020). 32570124 10.1016/j.jss.2020.05.039 PMC7303623

[27] Mermin-Bunnell, K. et al. Use of natural language processing of patient-initiated electronic health record messages to identify patients with COVID-19 infection. *JAMA Netw. Open* **6**, e2322299, 10.1001/jamanetworkopen.2023.22299 (2023). 37418261 10.1001/jamanetworkopen.2023.22299 PMC10329205

[28] Mastorakos, G. et al. Probing patient messages enhanced by natural language processing: a top-down message corpus analysis. *Health Data Sci*. 2021. 10.34133/2021/1504854. (2021) 10.34133/2021/1504854 PMC10877700 38487509

[29] Ko, D.G. et al. Beyond time-based billing: an ai-enabled complexity model for secure message reimbursement. *Telemed J E Health*. 10.1177/15305627251377376. (2025). 10.1177/15305627251377376 40984817

[30] McGeady, D., Kujala, J. & Ilvonen, K. The impact of patient–physician web messaging on healthcare service provision. *Int. J. Med. Inf.* **77**, 17–23, 10.1016/j.ijmedinf.2006.11.004 (2008). 10.1016/j.ijmedinf.2006.11.004 17188564

[31] Wallwiener, M. et al. Impact of electronic messaging on the patient-physician interaction. *J. Telemed. Telecare* **15**, 243–250, 10.1258/jtt.2009.090111 (2009). 19590030 10.1258/jtt.2009.090111

[32] Goldzweig, C.L. et al. Systematic review: secure messaging between providers and patients, and patients’ access to their own medical record: evidence on health outcomes, satisfaction, efficiency and attitudes. Washington (DC): Department of Veterans Affairs (US); (2012). 22973584

[33] Liu, X. et al. Evaluation of secure messaging applications for a health care system: a case study. *Appl. Clin. Inf.* **10**, 140–150, 10.1055/s-0039-1678607 (2019). 10.1055/s-0039-1678607 PMC6393161 30812040

[34] Kuo, A. & Dang, S. Secure messaging in electronic health records and its impact on diabetes clinical outcomes: a systematic review. *Telemed. E-Health* **22**, 769–777, 10.1089/tmj.2015.0207 (2016). 10.1089/tmj.2015.0207 27027337

[35] Wec, A. et al. Measurement, drivers, and outcomes of patient-initiated secure messaging use and intensity: a scoping review. *JAMIA Open* **8**, ooaf087, 10.1093/jamiaopen/ooaf087 (2025). 40799929 10.1093/jamiaopen/ooaf087 PMC12342875

[36] Conroy, M. et al. Electronic medical record–based electronic messaging among patients with breast cancer: a systematic review. *Appl. Clin. Inf.* **14**, 134–143, 10.1055/a-2004-6669 (2023). 10.1055/a-2004-6669 PMC9931493 36581054

[37] Hu, D. et al. A systematic review of early evidence on generative AI for drafting responses to patient messages. *Npj Health Syst.* **2**, 27, 10.1038/s44401-025-00032-5 (2025). 10.1038/s44401-025-00032-5 PMC13354203 42527471

[38] Guo, Y. et al. Computational use of patient–provider secure messaging data to achieve better clinical efficiency and quality of communication: a systematic review 10.1101/2025.05.19.25327936. (2025). PMC12919550 41726454

[39] Robinson, S. A. et al. Secure messaging, diabetes self-management, and the importance of patient autonomy: a mixed methods study. *J. Gen. Intern. Med.* **35**, 2955–2962, 10.1007/s11606-020-05834-x (2020). 32440998 10.1007/s11606-020-05834-x PMC7572993

[40] Harris, L. T. et al. Glycemic control associated with secure patient-provider messaging within a shared electronic medical record: a longitudinal analysis. *Diab. Care* **36**, 2726–2733, 10.2337/dc12-2003 (2013). 10.2337/dc12-2003 PMC3747898 23628618

[41] Russell, N.M. et al. Text-messaging to support diabetes self-management in a rural health clinic: a quality improvement project. *Online J. Nurs. Inform.* **21**, https://www.caremessage.org/text-messaging-to-support-diabetes-self-management/ (2017).

[42] Heisey-Grove, D. M. et al. Associations between patient health outcomes and secure message content exchanged between patients and clinicians: retrospective cohort study. *J. Med. Internet Res.* **22**, e19477, 10.2196/19477 (2020). 33118938 10.2196/19477 PMC7661231

[43] Price-Haywood, E. G., Luo, Q. & Monlezun, D. Dose effect of patient-care team communication via secure portal messaging on glucose and blood pressure control. *J. Am. Med. Inf. Assoc.* **25**, 702–708, 10.1093/jamia/ocx161 (2018). 10.1093/jamia/ocx161 PMC7647025 29444256

[44] Shimada, S. L. et al. Sustained use of patient portal features and improvements in diabetes physiological measures. *J. Med. Internet Res.* **18**, e179, 10.2196/jmir.5663 (2016). 27369696 10.2196/jmir.5663 PMC4947193

[45] Robinson, S. A. et al. Differences in secure messaging, self-management, and glycemic control between rural and urban patients: secondary data analysis. *JMIR Diab.* **6**, e32320, 10.2196/32320 (2021). 10.2196/32320 PMC8663667 34807834

[46] Lyles, C. R. et al. Communication about diabetes risk factors during between-visit encounters. *Am. J. Manag Care* **18**, 807–815 (2012). 23286610

[47] Quinn, C. C. et al. Mobile diabetes intervention study of patient engagement and impact on blood glucose: mixed methods analysis. *JMIR MHealth UHealth* **6**, e31, 10.2196/mhealth.9265 (2018). 29396389 10.2196/mhealth.9265 PMC5816260

[48] Bredfeldt, C. E., Compton-Phillips, A. L. & Snyder, M. H. Effects of between visit physician-patient communication on Diabetes Recognition Program scores. *Int. J. Qual. Health Care* **23**, 664–673, 10.1093/intqhc/mzr061 (2011). 21937586 10.1093/intqhc/mzr061

[49] Alexander, J. & Beatty, A. Association of patient portal messaging with survival among radiation oncology patients. *Int. J. Radiat. Oncol. Biol. Phys.* **120**, 627–638, 10.1016/j.ijrobp.2024.05.003 (2024). 38723754 10.1016/j.ijrobp.2024.05.003

[50] Wagner, L. I. et al. Bringing PROMIS to practice: brief and precise symptom screening in ambulatory cancer care. *Cancer* **121**, 927–934, 10.1002/cncr.29104 (2015). 25376427 10.1002/cncr.29104 PMC4352124

[51] Ralston, J. D. et al. Home blood pressure monitoring, secure electronic messaging and medication intensification for improving hypertension control: a mediation analysis. *Appl. Clin. Inf.* **5**, 232–248, 10.4338/ACI-2013-10-RA-0079 (2014). 10.4338/ACI-2013-10-RA-0079 PMC3974258 24734136

[52] Iturralde, E. et al. Changing results-engage and activate to enhance wellness: a randomized clinical trial to improve cardiovascular risk management. *J. Am. Heart Assoc.* **8**, e014021, 10.1161/JAHA.119.014021 (2019). 31787053 10.1161/JAHA.119.014021 PMC6912976

[53] North, F. et al. Telemonitoring blood pressure by secure message on a patient portal: use, content, and outcomes. *Telemed. J. E Health* **21**, 630–636, 10.1089/tmj.2014.0179 (2015). 25885765 10.1089/tmj.2014.0179

[54] Cutrona, S. L. et al. Improving rates of outpatient influenza vaccination through EHR portal messages and interactive automated calls: a randomized controlled trial. *J. Gen. Intern. Med.* **33**, 659–667, 10.1007/s11606-017-4266-9 (2018). 29383550 10.1007/s11606-017-4266-9 PMC5910339

[55] Wijesundara, J. G. et al. Electronic health record portal messages and interactive voice response calls to improve rates of early season influenza vaccination: randomized controlled trial. *J. Med. Internet Res.* **22**, e16373, 10.2196/16373 (2020). 32975529 10.2196/16373 PMC7547389

[56] Khan, A. A. et al. A learning health system approach to increasing human papillomavirus immunizations among young adults. *Perm. J.* **27**, 31–36, 10.7812/TPP/22.094 (2023). 37221889 10.7812/TPP/22.094 PMC10266845

[57] Ueberroth, B. E., Labonte, H. R. & Wallace, M. R. Impact of patient portal messaging reminders with self-scheduling option on influenza vaccination rates: a prospective, randomized trial. *J. Gen. Intern. Med.* **37**, 1394–1399, 10.1007/s11606-021-06941-z (2022). 34131878 10.1007/s11606-021-06941-z PMC8205315

[58] Berset, A. E. et al. Effect of electronic outreach using patient portal messages on well child care visit completion: a randomized clinical trial. *JAMA Netw. Open* **5**, e2242853, 10.1001/jamanetworkopen.2022.42853 (2022). 36399342 10.1001/jamanetworkopen.2022.42853 PMC9675005

[59] Rauhut, M. A. Digital engagement and the efficacy of patient portal-based preventive care interventions. *Digit Health* **11**, 20552076251356013, 10.1177/20552076251356013 (2025). 40585056 10.1177/20552076251356013 PMC12205184

[60] Goshgarian, G. et al. Effect of patient portal messaging before mailing fecal immunochemical test Kit on colorectal cancer screening rates: a randomized clinical trial. *JAMA Netw. Open* **5**, e2146863, 10.1001/jamanetworkopen.2021.46863 (2022). 35119462 10.1001/jamanetworkopen.2021.46863 PMC8817202

[61] Halket, D. et al. Targeted electronic patient portal messaging increases Hepatitis C virus screening in primary care: a randomized study. *J. Gen. Intern. Med.* **37**, 3318–3324, 10.1007/s11606-022-07460-1 (2022). 35230622 10.1007/s11606-022-07460-1 PMC9551157

[62] Hojat, L. et al. Doubling Hepatitis C virus screening in primary care using advanced electronic health record tools—a non-randomized controlled trial. *J. Gen. Intern. Med.* **35**, 498–504, 10.1007/s11606-019-05536-z (2020). 31792863 10.1007/s11606-019-05536-z PMC7018893

[63] Yedulla, N. R. et al. Pre-visit digital messaging improves patient-reported outcome measure participation prior to the orthopaedic ambulatory visit: results from a double-blinded, prospective, randomized controlled trial. *J. Bone Jt Surg. Am.* **105**, 20–26, 10.2106/JBJS.21.00506 (2023). 10.2106/JBJS.21.00506 36598473

[64] Yip, J.Y. et al. Impact of a patient portal-based telehealth outreach program on recall of patients with diabetic retinopathy. *Telemed E-Health*. 10.1089/tmj.2024.0454. (2024). 10.1089/tmj.2024.0454 39831324

[65] Lam, S. W. et al. A novel detection method to identify individuals with Alpha-1 Antitrypsin deficiency: linking prescription of COPD medications with the patient-facing electronic medical record. *Chronic Obstr. Pulm. Dis.* **9**, 26–33, 10.15326/jcopdf.2021.0260 (2022). 34784453 10.15326/jcopdf.2021.0260 PMC8893965

[66] Sawicki, C. et al. Two-way clinical messaging in a CML specialty pharmacy service model. *J. Manag. Care Spec. Pharm.* **25**, 1290–1296, 10.18553/jmcp.2019.25.11.1290 (2019). 31663460 10.18553/jmcp.2019.25.11.1290 PMC10397804

[67] Dicianno, B. E. et al. Feasibility of using mobile health to promote self-management in Spina Bifida. *Am. J. Phys. Med Rehabil.* **95**, 425–437, 10.1097/PHM.0000000000000400 (2016). 26488144 10.1097/PHM.0000000000000400

[68] Turvey, C. et al. Secure messaging intervention in patients starting new antidepressant to promote adherence: pilot randomized controlled trial. *JMIR Form. Res.* **7**, e51277, 10.2196/51277 (2023). 38064267 10.2196/51277 PMC10746966

[69] Manka, M. G. et al. Assessing the impact of hospital dismissal summary readability on patient outcomes following prostatectomy. *Urology* **157**, 201–205, 10.1016/j.urology.2021.06.040 (2021). 34303758 10.1016/j.urology.2021.06.040

[70] Tieu, C. et al. Utilization of patient electronic messaging to promote advance care planning in the primary care setting. *Am. J. Hosp. Palliat. Care* **34**, 665–670, 10.1177/1049909116650237 (2017). 27188759 10.1177/1049909116650237

[71] Ginting, K. et al. Patient portal, patient-generated images, and medical decision-making in a pediatric ambulatory setting. *Appl. Clin. Inf.* **11**, 764–768, 10.1055/s-0040-1718754 (2020). 10.1055/s-0040-1718754 PMC7673956 33207384

[72] Dang, S. et al. Evaluating an electronic health record intervention for management of heart failure among veterans. *Telemed. J. E Health* **24**, 1006–1013, 10.1089/tmj.2017.0307 (2018). 29672218 10.1089/tmj.2017.0307

[73] Lamba, A. H. et al. Characteristics of women enrolled in a patient portal intervention for menopause. *Women’s. Health Rep. N. Rochelle* **1**, 500–510, 10.1089/whr.2020.0091 (2020). 10.1089/whr.2020.0091 PMC7784774 33786517

[74] Sadasivam, R. S. et al. Secure asynchronous communication between smokers and tobacco treatment specialists: secondary analysis of a web-assisted tobacco intervention in the QUIT-PRIMO and National dental PBRN networks. *J. Med. Internet Res.* **22**, e13289, 10.2196/13289 (2020). 32374266 10.2196/13289 PMC7240437

[75] “Can you hear me now?”: postoperative patient-initiated communication with providers. *Am. J. Manag. Care* **26**, e333–e341. 10.37765/ajmc.2020.88507. (2020). 10.37765/ajmc.2020.88507 33094946

[76] Zurlo, J. et al. OPT-in for life: a mobile technology–based intervention to improve HIV care continuum for young adults living with HIV. *Health Promot Pract.* **21**, 727–737, 10.1177/1524839920936247 (2020). 32757835 10.1177/1524839920936247

[77] Diyaolu, M. et al. Outcome assessment of office Plastibell circumcision in infants utilizing interactive electronic health record. *J. Pediatr. Surg.* **58**, 1008–1013, 10.1016/j.jpedsurg.2023.01.043 (2023). 36797109 10.1016/j.jpedsurg.2023.01.043

[78] Smith, J. R. et al. High-frequency utilization of the outpatient messaging system in a specialized outpatient catatonia clinic for individuals with Autism spectrum disorder. *J. Child Adolesc. Psychopharmacol.* **35**, 424–430, 10.1089/cap.2025.0034 (2025). 40302606 10.1089/cap.2025.0034 PMC12570861

[79] Carlson, K. J. et al. The effect of geographic rounding on hospitalist work experience: a mixed-methods study. *Hosp. Pract.* **50**, 124–131, 10.1080/21548331.2022.2050649 (2022). 10.1080/21548331.2022.2050649 35253585

[80] Bala, T. R. An improvement project in reducing after-visit phone calls in a community pediatric neurology clinic: too much communication?. *Neurol. Clin. Pract.* **14**, e200269, 10.1212/CPJ.0000000000200269 (2024). 38516342 10.1212/CPJ.0000000000200269 PMC10955459

[81] Edwards, A. L. et al. Association between gastrointestinal symptoms and specialty care utilization among colon cancer survivors: a cohort study. *Int. J. Colorectal Dis.* **39**, 130, 10.1007/s00384-024-04685-w (2024). 39138736 10.1007/s00384-024-04685-w PMC11322428

[82] Powell, D. S. et al. The annual wellness visit health risk assessment: potential of patient portal-based completion and patient-oriented education and support. *Innov. Aging* **8**, igae023, 10.1093/geroni/igae023 (2024). 38618518 10.1093/geroni/igae023 PMC11010311

[83] Powell, K. R. & Deroche, C. Predictors and patterns of portal use in patients with multiple chronic conditions. *Chronic Illn.* **16**, 275–283, 10.1177/1742395318803663 (2020). 30284917 10.1177/1742395318803663

[84] Zhong, X. et al. The impact of e-visits on patient access to primary care. *Health Care Manag. Sci.* **21**, 475–491, 10.1007/s10729-017-9404-8 (2018). 28523477 10.1007/s10729-017-9404-8

[85] Liss, D. T. et al. Changes in office visit use associated with electronic messaging and telephone encounters among patients with diabetes in the PCMH. *Ann. Fam. Med.* **12**, 338–343, 10.1370/afm.1642 (2014). 25024242 10.1370/afm.1642 PMC4096471

[86] North, F. et al. Patient-generated secure messages and eVisits on a patient portal: are patients at risk?. *J. Am. Med. Inf. Assoc.* **20**, 1143–1149, 10.1136/amiajnl-2012-001208 (2013). 10.1136/amiajnl-2012-001208 PMC3822104 23703826

[87] Heisey-Grove, D. et al. Associations between patient-provider secure message content and patients’ health care visits. *Telemed. J. E Health* **28**, 690–698, 10.1089/tmj.2021.0164 (2022). 34569867 10.1089/tmj.2021.0164

[88] Plate, J. F. et al. Utilization of an electronic patient portal following total joint arthroplasty does not decrease readmissions. *J. Arthroplast.* **34**, 211–214, 10.1016/j.arth.2018.11.002 (2019). 10.1016/j.arth.2018.11.002 30497899

[89] Hong, Y. R. et al. Trends in e-visit adoption among U.S. office-based physicians: evidence from the 2011-2015 NAMCS. *Int. J. Med. Inf.* **129**, 260–266, 10.1016/j.ijmedinf.2019.06.025 (2019). 10.1016/j.ijmedinf.2019.06.025 31445265

[90] Neeman, E. et al. Attitudes and perceptions of multidisciplinary cancer care clinicians toward telehealth and secure messages. *JAMA Netw. Open* **4**, e2133877, 10.1001/jamanetworkopen.2021.33877 (2021). 34817586 10.1001/jamanetworkopen.2021.33877 PMC8613601

[91] Bavafa, H., Hitt, L. M. & Terwiesch, C. The impact of E-visits on visit frequencies and patient health: evidence from primary care. *Manag. Sci.* **64**, 5461–5480, 10.1287/mnsc.2017.2900 (2018). 10.1287/mnsc.2017.2900 PMC7540911 33033417

[92] Warren, C. J. et al. A Tale of 102,726 messages: characterizing the modern urologist’s portal message burden after common urologic surgeries. *Urol Pract.* 12. 10.1097/UPJ.0000000000000718. (2025). 10.1097/UPJ.0000000000000718 39383007

[93] Ozdag, Y., Makar, G. S. & Kolessar, D. J. Postoperative communication volume following total joint arthroplasty can be a precursor for emergency department visits. *Arthroplast Today* **27**, 101352, 10.1016/j.artd.2024.101352 (2024). 38690097 10.1016/j.artd.2024.101352 PMC11058096

[94] Chua, T.L. et al. Post-operative patient portal messaging is associated with return to the emergency department after lumbar spine surgery. *Spine*. 10.1097/BRS.0000000000005006. (2024). 10.1097/BRS.0000000000005006 38597199

[95] Hoonakker, P. L. T., Carayon, P. & Cartmill, R. S. The impact of secure messaging on workflow in primary care: results of a multiple-case, multiple-method study. *Int. J. Med. Inf.* **100**, 63–76, 10.1016/j.ijmedinf.2017.01.004 (2017). 10.1016/j.ijmedinf.2017.01.004 PMC8365630 28241939

[96] Shimada, S. L. et al. Patient-provider secure messaging in VA: variations in adoption and association with urgent care utilization. *Med. Care* **51**, S21–S28, 10.1097/MLR.0b013e3182780917 (2013). 23407007 10.1097/MLR.0b013e3182780917

[97] Reed, M. et al. Patient-initiated e-mails to providers: associations with out-of-pocket visit costs, and impact on care-seeking and health. *Am. J. Manag Care* **21**, e632–e639 (2015). 26760425

[98] O’Shea, A. M. J. et al. Association of secure messaging with primary care in-person and telephone visits among veterans: a matched difference-in-difference analysis. *J. Gen. Intern Med* **36**, 946–951, 10.1007/s11606-020-06541-3 (2021). 33528777 10.1007/s11606-020-06541-3 PMC8041942

[99] Kachroo, N. et al. Does “MyChart” Benefit “My” Surgery? A Look at the impact of electronic patient portals on patient experience. *J. Urol.* **204**, 760–768, 10.1097/JU.0000000000001090 (2020). 32330407 10.1097/JU.0000000000001090

[100] Apaydin, E. A. et al. Secure messages, video visits, and burnout among primary care providers in the Veterans Health Administration: national survey study. *J. Med. Internet Res.* **27**, e68858, 10.2196/68858 (2025). 40911914 10.2196/68858 PMC12413187

[101] Baxter, S. L. et al. Association of electronic health record in-basket message characteristics with physician burnout. *JAMA Netw. Open* **5**, e2244363, 10.1001/jamanetworkopen.2022.44363 (2022). 36449288 10.1001/jamanetworkopen.2022.44363 PMC9713605

[102] Pfaff, E. et al. Recruiting for a pragmatic trial using the electronic health record and patient portal: successes and lessons learned. *J. Am. Med. Inf. Assoc.* **26**, 44–49, 10.1093/jamia/ocy138 (2019). 10.1093/jamia/ocy138 PMC6308009 30445631

[103] Miller, H. N. et al. Electronic medical record-based cohort selection and direct-to-patient, targeted recruitment: early efficacy and lessons learned. *J. Am. Med. Inf. Assoc.* **26**, 1209–1217, 10.1093/jamia/ocz168 (2019). 10.1093/jamia/ocz168 PMC6798572 31553434

[104] Plante, T. B. et al. Recruitment of trial participants through electronic medical record patient portal messaging: a pilot study. *Clin.Trials* **17**, 30–38, 10.1177/1740774519873657 (2020). 31581836 10.1177/1740774519873657 PMC6992491

[105] Beaton, M. et al. Using patient portals for large-scale recruitment of individuals underrepresented in biomedical research: an evaluation of engagement patterns throughout the patient portal recruitment process at a single site within the All of Us Research Program. *J. Am. Med. Inf. Assoc.* **31**, 2328–2336, 10.1093/jamia/ocae135 (2024). 10.1093/jamia/ocae135 PMC11413441 38917428

[106] Miller, H. N. et al. Use of electronic recruitment methods in a clinical trial of adults with gout. *Clin. Trials* **18**, 92–103, 10.1177/1740774520956969 (2021). 32933342 10.1177/1740774520956969 PMC7878277

[107] Pogue, J. R. et al. Strategies and lessons learned from a longitudinal study to maximize recruitment in the midst of a global pandemic. *Proc. Bayl. Univ. Med. Cent.* **35**, 309–314, 10.1080/08998280.2022.2034494 (2022). 35518796 10.1080/08998280.2022.2034494 PMC9037400

[108] Nassif, M. et al. Recruitment strategies of a decentralized randomized placebo controlled clinical trial: the canagliflozin impact on health status, quality of life and functional status in heart failure (CHIEF-HF) Trial. *J. Card. Fail* **29**, 863–869, 10.1016/j.cardfail.2023.04.001 (2023). 37040839 10.1016/j.cardfail.2023.04.001

[109] Baucom, R. B. et al. Case report: patient portal versus telephone recruitment for a surgical research study. *Appl. Clin. Inf.* **5**, 1005–1014, 10.4338/ACI-2014-07-CR-0059 (2014). 10.4338/ACI-2014-07-CR-0059 PMC4287677 25589913

[110] Dykes, C. et al. Implementation of MyChart for recruitment at an academic medical center. *J. Clin. Transl. Sci.* **8**, e160, 10.1017/cts.2024.605 (2024). 39540113 10.1017/cts.2024.605 PMC11557278

[111] Ziegenfuss, J.Y. et al. A randomized study comparing patient portal and email communications for trial recruitment. *Clin. Trials* 17407745251358259. 10.1177/17407745251358259. (2025). 10.1177/17407745251358259 PMC12373006 40836901

[112] Peeler, A. et al. Centralized registry for COVID-19 research recruitment: design, development, implementation, and preliminary results. *J. Clin. Transl. Sci.* **5**, e152, 10.1017/cts.2021.819 (2021). 34462668 10.1017/cts.2021.819 PMC8387691

[113] Loftus, J. R. et al. Impact of early direct patient notification on follow-up completion for nonurgent actionable incidental radiologic findings. *J. Am. Coll. Radio.* **21**, 558–566, 10.1016/j.jacr.2023.07.026 (2024). 10.1016/j.jacr.2023.07.026 37820835

[114] Sun, S. et al. Messaging to your doctors: understanding patient-provider communications via a portal system, Paris, France: Association for Computing Machinery. 1739–1748. 10.1145/2470654.2466230. (2013).

[115] Mehta, S. J. et al. Behavioral interventions to improve population health outreach for hepatitis C screening: randomized clinical trial. *BMJ* **373**, n1022, 10.1136/bmj.n1022 (2021). 34006604 10.1136/bmj.n1022 PMC8129827

[116] Lieu, T. A. et al. Effect of electronic and mail outreach from primary care physicians for COVID-19 vaccination of black and latino older adults: a randomized clinical trial. *JAMA Netw. Open* **5**, e2217004, 10.1001/jamanetworkopen.2022.17004 (2022). 35713906 10.1001/jamanetworkopen.2022.17004 PMC9206195

[117] Chen, Y. et al. “I don’t bother with the phone!”. *Feeling Closer Phys. using Secur. Messaging* **2017**, 3813–3822 (2017).

[118] Greenwood, D. A. et al. A comparison of in-person, telephone, and secure messaging for Type 2 diabetes self-management support. *Diab. Educ.* **40**, 516–525, 10.1177/0145721714531337 (2014). 10.1177/0145721714531337 24742540

[119] Bundogji, N., Toma, G. & Khan, A. Identification of preferred reminder systems and patient factors to promote adherence in the management of urinary incontinence. *PEC Innov.* **1**, 100067, 10.1016/j.pecinn.2022.100067 (2022). 37213766 10.1016/j.pecinn.2022.100067 PMC10194242

[120] Paige, S. R. et al. Patient message preferences to promote clinical conversations about chronic obstructive pulmonary disease (COPD): a discrete choice experiment. *PEC Innov.* **2**, 100168, 10.1016/j.pecinn.2023.100168 (2023). 37384164 10.1016/j.pecinn.2023.100168 PMC10294043

[121] Raghu, T. S. et al. Using secure messaging to update medications list in ambulatory care setting. *Int. J. Med. Inf.* **84**, 754–762, 10.1016/j.ijmedinf.2015.06.003 (2015). 10.1016/j.ijmedinf.2015.06.003 26113460

[122] Whiteside, U. et al. Online cognitive behavioral therapy for depressed primary care patients: a pilot feasibility project. *Perm. J.* **18**, 21–27, 10.7812/TPP/13-155 (2014). 24867546 10.7812/TPP/13-155 PMC4022553

[123] Sequist, T. D. et al. Electronic patient messages to promote colorectal cancer screening: a randomized controlled trial. *Arch. Intern. Med.* **171**, 636–641, 10.1001/archinternmed.2010.467 (2011). 21149743 10.1001/archinternmed.2010.467 PMC3169179

[124] Lee, J. K. et al. Randomized trial of patient outreach approaches to de-implement outdated colonoscopy surveillance intervals. *Clin. Gastroenterol. Hepatol.* **22**, 1315–1322.e7, 10.1016/j.cgh.2023.12.027 (2024). 38191014 10.1016/j.cgh.2023.12.027

[125] Bennett, W. L. et al. Patient recruitment into a multicenter clinical cohort linking electronic health records from 5 health systems: cross-sectional analysis. *J. Med. Internet Res.* **23**, e24003, 10.2196/24003 (2021). 34042604 10.2196/24003 PMC8193474

[126] Fisher, L. et al. A novel household-based patient outreach pilot program to boost late-season influenza vaccination rates during the COVID-19 pandemic. *Influenza Other Respir. Viruses* **16**, 1141–1150, 10.1111/irv.13041 (2022). 36098249 10.1111/irv.13041 PMC9530505

[127] Rand, M. L. Nursing interventions increase influenza vaccination quality measures for home telehealth patients. *J. Nurs. Care Qual.* **37**, 47–53, 10.1097/NCQ.0000000000000577 (2022). 34224534 10.1097/NCQ.0000000000000577

[128] Szilagyi, P. G. et al. Text vs patient portal messaging to improve influenza vaccination coverage: a health system-wide randomized clinical trial. *JAMA Intern. Med.* **184**, 519–527, 10.1001/jamainternmed.2024.0001 (2024). 38497955 10.1001/jamainternmed.2024.0001 PMC10949147

[129] Haff, N. et al. “How” Versus “Why” messaging to increase uptake of booster vaccination against COVID-19: results of a pragmatic randomized trial. *J. Gen. Intern. Med.* **39**, 611–618, 10.1007/s11606-023-08492-x (2024). 37932539 10.1007/s11606-023-08492-x PMC10973315

[130] Szilagyi, P. G. et al. Video and infographic messages from primary care physicians and influenza vaccination rates: a randomized clinical trial. *JAMA Netw. Open* **8**, e2526514–e2526514, 10.1001/jamanetworkopen.2025.26514 (2025). 40802184 10.1001/jamanetworkopen.2025.26514 PMC12351418

[131] Houston, T. K. et al. Evaluating the QUIT-PRIMO clinical practice ePortal to increase smoker engagement with online cessation interventions: a national hybrid type 2 implementation study. *Implement Sci.* **10**, 154, 10.1186/s13012-015-0336-8 (2015). 26525410 10.1186/s13012-015-0336-8 PMC4630887

[132] Erdmann, M., Edwards, B. & Adewumi, M. T. Effect of electronic portal messaging with embedded asynchronous care on physician-assisted smoking cessation attempts: a randomized clinical trial. *JAMA Netw. Open* **5**, e220348, 10.1001/jamanetworkopen.2022.0348 (2022). 35226082 10.1001/jamanetworkopen.2022.0348 PMC8886534

[133] Midboe, A. M. et al. Relationship between patient portal tool use and medication adherence and viral load among patients living with HIV. *J. Gen. Intern. Med.* **39**, 127–135, 10.1007/s11606-023-08474-z (2024). 38252241 10.1007/s11606-023-08474-z PMC10937883

[134] McInnes, D. K. et al. Patient use of electronic prescription refill and secure messaging and its association with undetectable HIV viral load: a retrospective cohort study. *J. Med. Internet Res.* **19**, e34, 10.2196/jmir.6932 (2017). 28202428 10.2196/jmir.6932 PMC5332835

[135] Nagler, A.R. et al. Patient portal messaging to address delayed follow-up for uncontrolled diabetes: a pragmatic, randomised clinical trial. *BMJ Qual Saf.*. 10.1136/bmjqs-2024-018249. (2025). 10.1136/bmjqs-2024-018249 40348403

[136] Ralston, J. D. et al. Use of web-based shared medical records among patients with HIV. *Am. J. Manag Care* **19**, e114–e124 (2013). 23725449 PMC3951974

[137] Steitz, B. et al. Long-term patterns of patient portal use for pediatric patients at an academic medical center. *Appl. Clin. Inf.* **8**, 779–793, 10.4338/ACI-2017-01-RA-0005 (2017). 10.4338/ACI-2017-01-RA-0005 PMC6220688 28765865

[138] Waselewski, M. E. et al. A mobile health app to support patients receiving medication-assisted treatment for opioid use disorder: development and feasibility study. *JMIR Form. Res.* **5**, e24561, 10.2196/24561 (2021). 33620324 10.2196/24561 PMC7943342

[139] Yamin, C. K. et al. The digital divide in adoption and use of a personal health record. *Arch. Intern. Med.* **171**, 568–574, 10.1001/archinternmed.2011.34 (2011). 21444847 10.1001/archinternmed.2011.34

[140] Hefner, J. L. et al. Patient portal messaging for care coordination: a qualitative study of perspectives of experienced users with chronic conditions. *BMC Fam. Pract.* **20**, 57, 10.1186/s12875-019-0948-1 (2019). 31053063 10.1186/s12875-019-0948-1 PMC6499960

[141] Furukawa, M. F. et al. WEB FIRST. Despite substantial progress in ehr adoption, health information exchange and patient engagement remain low in office settings. *Health Aff.* **33**, 1672–1679, 10.1377/hlthaff.2014.0445 (2014). 10.1377/hlthaff.2014.0445 25104827

[142] Cronin, R. M. et al. Growth of secure messaging through a patient portal as a form of outpatient interaction across clinical specialties. *Appl. Clin. Inf.* **6**, 288–304, 10.4338/ACI-2014-12-RA-0117 (2015). 10.4338/ACI-2014-12-RA-0117 PMC4493331 26171076

[143] Senft, N., Butler, E. & Everson, J. Growing disparities in patient-provider messaging: trend analysis before and after supportive policy. *J. Med. Internet Res.* **21**, e14976, 10.2196/14976 (2019). 31593539 10.2196/14976 PMC6803888

[144] Heisey-Grove, D. M. & Carretta, H. J. Disparities in secure messaging uptake between patients and physicians: longitudinal analysis of two national cross-sectional surveys. *J. Med. Internet Res.* **22**, e12611, 10.2196/12611 (2020). 32356775 10.2196/12611 PMC7229528

[145] Yousef, C. C. et al. Predicting health care providers’ acceptance of a personal health record secure messaging feature. *Appl. Clin. Inf.* **13**, 148–160, 10.1055/s-0041-1742217 (2022). 10.1055/s-0041-1742217 PMC8828451 35139562

[146] Masterman, M. et al. Adoption of secure messaging in a patient portal across pediatric specialties. *AMIA Annu. Symp. Proc.* **2016**, 1930–1939 (2016). 28269952 PMC5333207

[147] Shenson, J. A. et al. Rapid growth in surgeons’ use of secure messaging in a patient portal. *Surg. Endosc.* **30**, 1432–1440, 10.1007/s00464-015-4347-y (2016). 26123340 10.1007/s00464-015-4347-y PMC4881849

[148] North, F. et al. A retrospective analysis of provider-to-patient secure messages: how much are they increasing, who is doing the work, and is the work happening after hours? JMIR. *Med. Inf.* **8**, e16521, 10.2196/16521 (2020). 10.2196/16521 PMC7381047 32673238

[149] Haun, J. N. et al. Primary care virtual resource use prior and post COVID-19 pandemic onset. *BMC Health Serv. Res.* **22**, 1370. 10.1186/s12913-022-08790-w (2022). 36401239 10.1186/s12913-022-08790-w PMC9673210

[150] Martinez, K. A. et al. Patient portal message volume and time spent on the EHR: an observational study of primary care clinicians. *J. Gen. Intern. Med.* **39**, 566–572, 10.1007/s11606-023-08577-7 (2024). 38129617 10.1007/s11606-023-08577-7 PMC10973312

[151] Crotty, B. H. et al. Patient-to-physician messaging: volume nearly tripled as more patients joined system, but per capita rate plateaued. *Health Aff. Millwood* **33**, 1817–1822, 10.1377/hlthaff.2013.1145 (2014). 25288428 10.1377/hlthaff.2013.1145 PMC4418542

[152] Ryan, S.P. et al. The contemporary “in-basket”: an explosion of unchecked administrative expectations. *J. Arthroplasty*10.1016/j.arth.2025.06.069. (2025). 10.1016/j.arth.2025.06.069 40570989

[153] Arndt, B. G. et al. More tethered to the EHR: EHR workload trends among academic primary care physicians, 2019-2023. *Ann. Fam. Med.* **22**, 12–18, 10.1370/afm.3047 (2024). 38253499 10.1370/afm.3047 PMC11233089

[154] Huang, M. et al. Patient portal messaging for asynchronous virtual care during the COVID-19 pandemic: retrospective analysis. *JMIR Hum. Factors* **9**, e35187, 10.2196/35187 (2022). 35171108 10.2196/35187 PMC9084445

[155] Campbell, B. R. et al. PositiveLinks and the COVID-19 response: importance of low-barrier messaging for PLWH in non-urban virginia in a crisis. *AIDS Behav.* **25**, 3519–3527, 10.1007/s10461-021-03294-w (2021). 33974168 10.1007/s10461-021-03294-w PMC8111858

[156] Brickson, C. et al. Characterizing electronic messaging use among hospitalists and its association with patient volumes. *J. Hosp. Med.*. 10.1002/jhm.13462. (2024). 10.1002/jhm.13462 39033420

[157] Ko, S. A. et al. Secure messaging use among patients with depression: an analysis using real-world data. *Telemed. J. E Health* **30**, 2157–2164, 10.1089/tmj.2024.0171 (2024). 38916859 10.1089/tmj.2024.0171

[158] Hansen, M. A. et al. Impact of COVID-19 lockdown on patient-provider electronic communications. *J. Telemed. Telecare* **30**, 1285–1292, 10.1177/1357633X221146810 (2024). 36659875 10.1177/1357633X221146810 PMC9892807

[159] Holmgren, A. J. et al. National trends in oncology specialists’ EHR inbox work, 2019-2022. *J. Natl. Cancer Inst.* **117**, 1253–1259, 10.1093/jnci/djaf052 (2025). 40037649 10.1093/jnci/djaf052 PMC12145914

[160] Holmgren, A. J. et al. Assessing the impact of the COVID-19 pandemic on clinician ambulatory electronic health record use. *J. Am. Med. Inf. Assoc.* **29**, 453–460, 10.1093/jamia/ocab268 (2022). 10.1093/jamia/ocab268 PMC8689796 34888680

[161] Holmgren, A. J. et al. Changes in physician electronic health record use with the expansion of telemedicine. *JAMA Intern. Med.* **183**, 1357–1365, 10.1001/jamainternmed.2023.5738 (2023). 37902737 10.1001/jamainternmed.2023.5738 PMC10616769

[162] Smith, D. C. et al. Sudden shift to Telehealth in COVID-19: a retrospective cohort study of disparities in use of telehealth for prenatal care in a large midwifery service. *J. Midwifery Women’s. Health* **69**, 522–530, 10.1111/jmwh.13601 (2024). 38111228 10.1111/jmwh.13601 PMC11182882

[163] Shimada, S. L. et al. Personal health record reach in the Veterans Health Administration: a cross-sectional analysis. *J. Med. Internet Res.* **16**, e272, 10.2196/jmir.3751 (2014). 25498515 10.2196/jmir.3751 PMC4275468

[164] Lyles, C. R. et al. Patient race/ethnicity and shared medical record use among diabetes patients. *Med. Care* **50**, 434–440, 10.1097/MLR.0b013e318249d81b (2012). 22354209 10.1097/MLR.0b013e318249d81b

[165] Riera, K. M. et al. Care delivered by pediatric surgical specialties through patient portal messaging. *J. Surg. Res.* **234**, 231–239, 10.1016/j.jss.2018.09.013 (2019). 30527479 10.1016/j.jss.2018.09.013 PMC6294474

[166] Weppner, W. G. et al. Use of a shared medical record with secure messaging by older patients with diabetes. *Diab. Care* **33**, 2314–2319, 10.2337/dc10-1124 (2010). 10.2337/dc10-1124 PMC2963486 20739686

[167] Neeman, E. et al. Future of teleoncology: trends and disparities in telehealth and secure message utilization in the COVID-19 Era. *JCO Clin. Cancer Inf.* **6**, e2100160, 10.1200/CCI.21.00160 (2022). 10.1200/CCI.21.00160 PMC9067360 35467963

[168] Chivela, F. L., Burch, A. E. & Asagbra, O. An assessment of patient portal messaging use by patients with multiple chronic conditions living in rural communities: retrospective analysis. *J. Med. Internet Res.* **25**, e44399, 10.2196/44399 (2023). 37526967 10.2196/44399 PMC10427930

[169] Huang, M. et al. Characterizing patient-clinician communication in secure medical messages: retrospective study. *J. Med. Internet Res.* **24**, e17273, 10.2196/17273 (2022). 35014964 10.2196/17273 PMC8790696

[170] Ralston, J. D. et al. Patient use of secure electronic messaging within a shared medical record: a cross-sectional study. *J. Gen. Intern. Med.* **24**, 349–355, 10.1007/s11606-008-0899-z (2009). 19137379 10.1007/s11606-008-0899-z PMC2642567

[171] Khalil, N. et al. Multiple sclerosis and MyChart messaging: a retrospective chart review evaluating its use. *Int. J. MS Care* **24**, 271–274, 10.7224/1537-2073.2020-101 (2022). 36545652 10.7224/1537-2073.2020-101 PMC9749828

[172] Mueller, B. et al. A retrospective cohort study of clinical factors, visit patterns, and demographic factors associated with use of remote communications in patients with headache. *Headache* **61**, 1521–1528, 10.1111/head.14226 (2021). 34713896 10.1111/head.14226

[173] Couture, A. & Birstler, J. The gender of the sender: assessing gender biases of greetings in patient portal messages. *J. Women’s. Health Larchmt.* **32**, 171–177, 10.1089/jwh.2022.0333 (2023). 36459624 10.1089/jwh.2022.0333 PMC10081704

[174] Bryan, M. et al. Resource utilization among portal users who send messages: a retrospective cohort study. *WMJ* **119**, 26–32 (2020). 32348068

[175] Yakushi, J. et al. Utilization of secure messaging to primary care departments. *Perm J.* **24**. 10.7812/TPP/19.177. (2020). 10.7812/TPP/19.177 PMC7213380 33196426

[176] Huang, M. et al. Characterizing the users of patient portal messaging: a single institutional cohort study. 381–387. 10.1109/ICHI57859.2023.00057. (2023).

[177] Fleming, N. L., Cullen, D. & Luna, G. An evaluation of patient web portal engagement: an exploratory study of patients with hypertension and diabetes. *Online J. Nurs. Inform.* **19**, 1–8 (2015).

[178] Monestime, J. P. et al. Characteristics of office-based providers associated with secure electronic messaging use: achieving meaningful use. *Int. J. Med. Inf.* **129**, 43–48, 10.1016/j.ijmedinf.2019.04.002 (2019). 10.1016/j.ijmedinf.2019.04.002 31445287

[179] Hansen, M. A. et al. Demographics and clinical features associated with rates of electronic message utilization in the primary care setting. *Int. J. Med. Inf.* **183**, 105339, 10.1016/j.ijmedinf.2024.105339 (2024). 10.1016/j.ijmedinf.2024.105339 38219417

[180] Siddiqui, S. et al. Use of the Veterans Health Administration online patient portal among Veterans with spinal cord injuries and disorders. *J. Spinal Cord. Med.* **46**, 917–928, 10.1080/10790268.2022.2084967 (2023). 35763563 10.1080/10790268.2022.2084967 PMC10653767

[181] Ukoha, E. P., Feinglass, J. & Yee, L. M. Disparities in electronic patient portal use in prenatal care: retrospective cohort study. *J. Med. Internet Res.* **21**, e14445, 10.2196/14445 (2019). 31586367 10.2196/14445 PMC6818527

[182] Levine, D. M., Linder, J. A. & Landon, B. E. Characteristics and disparities among primary care practices in the United States. *J. Gen. Intern. Med.* **33**, 481–486, 10.1007/s11606-017-4239-z (2018). 29204975 10.1007/s11606-017-4239-z PMC5880758

[183] Huang, M. et al. Midwest rural-urban disparities in use of patient online services for COVID-19. *J. Rural Health* **38**, 908–915, 10.1111/jrh.12657 (2022). 35261092 10.1111/jrh.12657 PMC9115171

[184] Graetz, I. et al. The digital divide and patient portals: internet access explained differences in patient portal use for secure messaging by age, race, and income. *Med. Care* **54**, 772–779, 10.1097/MLR.0000000000000560 (2016). 27314262 10.1097/MLR.0000000000000560

[185] Corriveau, B. et al. Sociodemographic variation in use of and preferences for digital technologies among patients in primary care: results from the OurCare national survey. *Can. Fam. Phys.* **71**, 324–336, 10.46747/cfp.7105324 (2025). 10.46747/cfp.7105324 PMC12087552 40368619

[186] Avoundjian, T. et al. Correlates of personal health record registration and utilization among veterans with HIV. *JAMIA Open* **4**, ooab029, 10.1093/jamiaopen/ooab029 (2021). 34278241 10.1093/jamiaopen/ooab029 PMC8280933

[187] Staloff, J. et al. High users of primary care secure messaging in the veterans health administration. *Ann. Fam. Med.* **23**, 285–293, 10.1370/afm.240360 (2025). 40721336 10.1370/afm.240360 PMC12306986

[188] Semere, W. et al. Secure messaging with physicians by proxies for patients with diabetes: findings from the ECLIPPSE Study. *J. Gen. Intern Med* **34**, 2490–2496, 10.1007/s11606-019-05259-1 (2019). 31428986 10.1007/s11606-019-05259-1 PMC6848304

[189] Alexander, J. & Beatty, A. L. Disparities in patient portal messaging among oncology patients enrolled in the patient portal. *JCO Clin. Cancer Inf.* **9**, e2400234, 10.1200/CCI-24-00234 (2025). 10.1200/CCI-24-00234 40632947

[190] Smith, S. G. et al. Disparities in registration and use of an online patient portal among older adults: findings from the LitCog cohort. *J. Am. Med. Inf. Assoc.* **22**, 888–895, 10.1093/jamia/ocv025 (2015). 10.1093/jamia/ocv025 PMC4810779 25914099

[191] Blok, A. C. et al. Impact of patient access to online VA notes on healthcare utilization and clinician documentation: a retrospective Cohort study. *J. Gen. Intern. Med.* **36**, 592–599, 10.1007/s11606-020-06304-0 (2021). 33443693 10.1007/s11606-020-06304-0 PMC7947092

[192] Harris, L. T. et al. Diabetes quality of care and outpatient utilization associated with electronic patient-provider messaging: A cross-sectional analysis. *Diab. Care* **32**, 1182–1187, 10.2337/dc08-1771 (2009). 10.2337/dc08-1771 PMC2699712 19366959

[193] Zhong, L. et al. Importance of prior patient interactions with the healthcare system to engaging with pretest cancer genetic services via digital health tools among unaffected primary care patients: findings from the BRIDGE Trial. *Health Serv. Res.* e14652. 10.1111/1475-6773.14652. (2025). 10.1111/1475-6773.14652 PMC12782189 40497580

[194] Steitz, B. D. et al. Repeated access to patient portal while awaiting test results and patient-initiated messaging. *JAMA Netw. Open* **8**, e254019–e254019, 10.1001/jamanetworkopen.2025.4019 (2025). 40198070 10.1001/jamanetworkopen.2025.4019 PMC11979724

[195] Hoopes, A.J. et al. Characteristics of adolescents who use secure messaging on a health system’s patient portal. *Pediatrics* 152. 10.1542/peds.2022-060271. (2023). 10.1542/peds.2022-060271 PMC10312232 37271795

[196] Ko, D-G. The impact of secure messaging telehealth service on the quality of healthcare. *Telehealth Med. Today* **8**. 10.30953/thmt.v8.435. (2023).

[197] Crotty, B. H. et al. Prevalence and risk profile of unread messages to patients in a patient web portal. *Appl. Clin. Inf.* **06**, 375–382, 10.4338/ACI-2015-01-CR-0006 (2015). 10.4338/ACI-2015-01-CR-0006 PMC4493337 26171082

[198] Tang, M. et al. Differences in care team response to patient portal messages by patient race and ethnicity. *JAMA Netw. Open* **7**, e242618, 10.1001/jamanetworkopen.2024.2618 (2024). 38497963 10.1001/jamanetworkopen.2024.2618 PMC10949096

[199] Jones, A. L. et al. Low uptake of secure messaging among veterans with experiences of homelessness and substance use disorders. *J. Addict. Med.* **15**, 508–511, 10.1097/ADM.0000000000000785 (2021). 33323688 10.1097/ADM.0000000000000785 PMC8200366

[200] Leung, L. B. et al. Characteristics of veterans experiencing homelessness using telehealth for primary care before and after COVID-19 pandemic onset. *J. Gen. Intern. Med.* **39**, 53–59, 10.1007/s11606-023-08462-3 (2024). 38252239 10.1007/s11606-023-08462-3 PMC10937850

[201] Holder, K. et al. Use of electronic patient messaging by pregnant patients receiving prenatal care at an academic health system: retrospective cohort study. *JMIR Mhealth Uhealth* **12**, e51637, 10.2196/51637 (2024). 38686560 10.2196/51637 PMC11146248

[202] Gordon, N. P. et al. Lower use of and potential barriers to using patient portals among limited english proficient latino and Chinese American adults: a health Techquity Concern. *Perm. J.* **29**, 1–22, 10.7812/TPP/24.119 (2025). 39935330 10.7812/TPP/24.119 PMC11907665

[203] Mora, J. et al. Language disparities in patient portal access and use among radiation oncology patients across an integrated health enterprise. *Int. J. Radiat. Oncol. Biol. Phys.* **120**, E48–E49 (2024).

[204] Roy, M. et al. Limited english proficiency and disparities in health care engagement among patients with breast cancer. *JCO Oncol. Pract.* **17**, e1837–e1845, 10.1200/OP.20.01093 (2021). 33844591 10.1200/OP.20.01093 PMC9810131

[205] Judson, T. J. et al. Patient Perceptions of e-visits: qualitative study of older adults to inform health system implementation. *JMIR Aging* **6**, e45641, 10.2196/45641 (2023). 37234031 10.2196/45641 PMC10257108

[206] Javier, S. J. et al. Racial and ethnic disparities in use of a personal health record by veterans living with HIV. *J. Am. Med. Inf. Assoc.* **26**, 696–702, 10.1093/jamia/ocz024 (2019). 10.1093/jamia/ocz024 PMC7647203 30924875

[207] Arizmendi, B. J. et al. Engagement in GI behavioral health is associated with reduced portal messages, phone calls, and ED visits. *Dig. Dis. Sci.* **69**, 1939–1947, 10.1007/s10620-024-08428-3 (2024). 38622464 10.1007/s10620-024-08428-3

[208] Wolcott, V., Agarwal, R. & Nelson, D. A. Is Provider secure messaging associated with patient messaging behavior? Evidence from the US Army. *J. Med. Internet Res.* **19**, e103, 10.2196/jmir.6804 (2017). 28385681 10.2196/jmir.6804 PMC5399218

[209] Heisey-Grove, D. et al. Patient and clinician characteristics associated with secure message content: retrospective Cohort study. *J. Med. Internet Res.* **23**, e26650, 10.2196/26650 (2021). 34420923 10.2196/26650 PMC8414300

[210] Branford, G. L. et al. The Gender Gap in EHR Workload: A Comparative Analysis of Primary Care Physician In Basket Usage. *J. Gen. Intern Med* **40**, 2255–2264, 10.1007/s11606-025-09629-w (2025). 40439865 10.1007/s11606-025-09629-w PMC12344033

[211] Ngo, M. et al. Gender based differences in electronic medical record utilization in an academic ophthalmology practice. *AJO Int.* **1**. 10.1016/j.ajoint.2024.100082. (2024).

[212] Rittenberg, E., Liebman, J. B. & Rexrode, K. M. Primary care physician gender and electronic health record workload. *J. Gen. Intern. Med.* **37**, 3295–3301, 10.1007/s11606-021-07298-z (2022). 34993875 10.1007/s11606-021-07298-z PMC9550938

[213] Scholes, J. et al. Special topic burnout: the digital workload divide: investigating gender differences in EHR messaging among primary care clinicians. *Appl. Clin. Inf*. 10.1055/a-2618-4580. (2025). 10.1055/a-2618-4580 PMC12513775 40404141

[214] Ha, D. et al. Gender differences in electronic health record inbox message volumes among pediatric surgeon specialists. *J. Pediatr. Surg.* **60**, 162191, 10.1016/j.jpedsurg.2025.162191 (2025). 39904266 10.1016/j.jpedsurg.2025.162191

[215] Haun, J. N. et al. Evaluating user experiences of the secure messaging tool on the Veterans Affairs’ patient portal system. *J. Med. Internet Res.* **16**, e75, 10.2196/jmir.2976 (2014). 24610454 10.2196/jmir.2976 PMC3961805

[216] Lam, R. et al. Older adult consumers’ attitudes and preferences on electronic patient-physician messaging. *Am. J. Manag. Care* **19**, eSP7–eSP11 (2013). 24511886 PMC4056337

[217] Paige, S. R. et al. Cancer patients’ satisfaction with telehealth during the COVID-19 pandemic. *PLoS ONE* **17**, e0268913, 10.1371/journal.pone.0268913 (2022). 35657778 10.1371/journal.pone.0268913 PMC9165798

[218] Kudel, I. & Perry, T. Communicating treatment-related symptoms using passively collected data and satisfaction/loyalty ratings: exploratory study. *JMIR Cancer* **8**, e29292, 10.2196/29292 (2022). 35175206 10.2196/29292 PMC9107057

[219] Short-Russell, M., Thompson, J. & Waldrop, J. Secure messaging: demonstration and enrollment patient portal program: patient portal use in vulnerable populations. *Comput. Inf. Nurs.* **42**, 104–108, 10.1097/CIN.0000000000001098 (2024). 10.1097/CIN.0000000000001098 38206326

[220] Heyworth, L. et al. Aligning medication reconciliation and secure messaging: qualitative study of primary care providers’ perspectives. *J. Med. Internet Res.* **15**, e264, 10.2196/jmir.2793 (2013). 24297865 10.2196/jmir.2793 PMC3868963

[221] Turvey, C. L. et al. Patient and provider experience of electronic patient portals and secure messaging in mental health treatment. *Telemed. J. E Health* **28**, 189–198, 10.1089/tmj.2020.0395 (2022). 33887164 10.1089/tmj.2020.0395 PMC8941946

[222] Haun, J. N. et al. Large-scale survey findings inform patients’ experiences in using secure messaging to engage in patient-provider communication and self-care management: a quantitative assessment. *J. Med. Internet Res.* **17**, e282, 10.2196/jmir.5152 (2015). 26690761 10.2196/jmir.5152 PMC4704939

[223] Rief, J. J. et al. Using health information technology to foster engagement: patients’ experiences with an active patient health record. *Health Commun.* **32**, 310–319, 10.1080/10410236.2016.1138378 (2017). 27223684 10.1080/10410236.2016.1138378 PMC10355811

[224] Brady, J. E. et al. The perceived effectiveness of secure messaging for medication reconciliation during transitions of care: semistructured interviews with patients. *JMIR Hum. Factors* **9**, e36652, 10.2196/36652 (2022). 35921139 10.2196/36652 PMC9386577

[225] Lieu, T. A. et al. Primary care physicians’ experiences with and strategies for managing electronic messages. *JAMA Netw. Open* **2**, e1918287, 10.1001/jamanetworkopen.2019.18287 (2019). 31880798 10.1001/jamanetworkopen.2019.18287 PMC6991215

[226] Harary, M. et al. Audit of postoperative readmissions and patient messages following endoscopic transnasal transsphenoidal surgery. *J. Neurol. Surg. B Skull Base* **83**, 611–617, 10.1055/a-1840-9874 (2022). 36393879 10.1055/a-1840-9874 PMC9653286

[227] Makhnoon, S. et al. Electronic patient portals as a modality for returning reclassified genetic test results. *Mol. Genet Genom. Med.* **13**, e70123, 10.1002/mgg3.70123 (2025). 10.1002/mgg3.70123 PMC12277930 40686326

[228] Mervak, B. M. et al. What the patient wants: an analysis of radiology-related inquiries from a web-based patient portal. *J. Am. Coll. Radio.* **13**, 1311–1318, 10.1016/j.jacr.2016.05.022 (2016). 10.1016/j.jacr.2016.05.022 27451118

[229] Heukelom, J. V. et al. Patient satisfaction with return of pharmacogenomic results utilizing a patient portal message. *Pharmacogenomics* **24**, 315–323, 10.2217/pgs-2023-0032 (2023). 37125619 10.2217/pgs-2023-0032 PMC10318570

[230] Wade-Vuturo, A. E., Mayberry, L. S. & Osborn, C. Y. Secure messaging and diabetes management: experiences and perspectives of patient portal users. *J. Am. Med. Inf. Assoc.* **20**, 519–525, 10.1136/amiajnl-2012-001253 (2013). 10.1136/amiajnl-2012-001253 PMC3628058 23242764

[231] Hoopes, A. J. et al. Teen secure messaging is associated with use of sexual and reproductive health services in one health system. *J. Adolesc. Health* **76**, 455–462, 10.1016/j.jadohealth.2024.10.016 (2025). 39580732 10.1016/j.jadohealth.2024.10.016 PMC12834321

[232] Haun, J. N. et al. Clinical practice informs secure messaging benefits and best practices. *Appl. Clin. Inf.* **8**, 1003–1011, 10.4338/ACI-2017-05-RA-0088 (2017). 10.4338/ACI-2017-05-RA-0088 PMC5802310 29241240

[233] Alpert, J. M. et al. Improving secure messaging: a framework for support, partnership & information-giving communicating electronically (SPICE). *Patient Educ. Couns.* **104**, 1380–1386, 10.1016/j.pec.2020.11.021 (2021). 33280967 10.1016/j.pec.2020.11.021

[234] Hernandez, B. F. et al. Communication preferences and satisfaction of secure messaging among patients and providers in the military healthcare system. *Mil. Med.* **183**, e383–e390, 10.1093/milmed/usy094 (2018). 29741659 10.1093/milmed/usy094

[235] Nazi, K. M. The personal health record paradox: health care professionals’ perspectives and the information ecology of personal health record systems in organizational and clinical settings. *J. Med. Internet Res.* **15**, e70, 10.2196/jmir.2443 (2013). 23557596 10.2196/jmir.2443 PMC3636319

[236] Brown, W. 3rd et al. Challenges and solutions to employing natural language processing and machine learning to measure patients’ health literacy and physician writing complexity: the ECLIPPSE study. *J. Biomed. Inf.* **113**, 103658, 10.1016/j.jbi.2020.103658 (2021). 10.1016/j.jbi.2020.103658 PMC8186847 33316421

[237] Eschler, J. et al. Designing asynchronous communication tools for optimization of patient-clinician coordination. *AMIA Annu. Symp. Proc.* **2015**, 543–552 (2015). 26958188 PMC4765629

[238] Stewart, M. T. et al. The promise of patient portals for individuals living with chronic illness: qualitative study identifying pathways of patient engagement. *J. Med. Internet Res.* **22**, e17744, 10.2196/17744 (2020). 32706679 10.2196/17744 PMC7395248

[239] Sieck, C. J. et al. The rules of engagement: perspectives on secure messaging from experienced ambulatory patient portal users. *MMIR Red Inf* **5**, e13, 10.2196/medinform.7516 (2017). 10.2196/medinform.7516 PMC5516097 28676467

[240] Bosold, A. L. et al. Older adults’ personal health information management: the role and perspective of various healthcare providers. *AMIA Annu. Symp. Proc.* **2021**, 255–264 (2021). 35308942 PMC8861717

[241] Alpert, J. M. et al. Clinicians’ attitudes and behaviors towards communicating electronically with patients: a grounded practical theory approach. *J. Health Commun.* **27**, 103–114, 10.1080/10810730.2022.2059723 (2022). 35380099 10.1080/10810730.2022.2059723

[242] Strand, M. et al. Exploring working relationships in mental health care via an E-recovery portal: qualitative study on the experiences of service users and health providers. *JMIR Ment. Health* **4**, e54, 10.2196/mental.8491 (2017). 29138127 10.2196/mental.8491 PMC5705858

[243] Haun, J. N. et al. Evaluating secure messaging from the veteran perspective: informing the adoption and sustained use of a patient-driven communication platform. *Ann. Anthropol. Pract.* **37**, 57–74, 10.1111/napa.12029 (2013).

[244] Knees, M. et al. Academic hospitalist perspectives on the benefits and challenges of secure messaging: a mixed methods analysis. *J. Hosp. Med.* **20**, 248–257, 10.1002/jhm.13522 (2025). 39358988 10.1002/jhm.13522

[245] King, G. et al. Connecting families to their health record and care team: the use, utility, and impact of a client/family health portal at a children’s rehabilitation hospital. *J. Med. Internet Res.* **19**, e97, 10.2196/jmir.6811 (2017). 28385680 10.2196/jmir.6811 PMC5399217

[246] Crotty, B. H., Mostaghimi, A. & Landon, B. E. Preparing residents for future practice: report of a curriculum for electronic patient-doctor communication. *Postgrad. Med. J.* **89**, 554–559, 10.1136/postgradmedj-2012-131688 (2013). 23680999 10.1136/postgradmedj-2012-131688

[247] Lew, D. et al. Association of EHR-integrated secure messaging use with clinician workload and attention switching. *J. Gen. Intern. Med*. 10.1007/s11606-025-09466-x. (2025). 10.1007/s11606-025-09466-x PMC12343377 40085321

[248] Akbar, F. et al. Physicians’ electronic inbox work patterns and factors associated with high inbox work duration. *J. Am. Med. Inf. Assoc.* **28**, 923–930, 10.1093/jamia/ocaa229 (2021). 10.1093/jamia/ocaa229 PMC8068414 33063087

[249] Laccetti, A. L. et al. Increase in cancer center staff effort related to electronic patient portal use. *J. Oncol. Pract.* **12**, e981–e990, 10.1200/JOP.2016.011817 (2016). 27601511 10.1200/JOP.2016.011817 PMC5455586

[250] Apathy, N. C. et al. Inbox message prioritization and management approaches in primary care. *JAMIA Open* **7**, ooae135, 10.1093/jamiaopen/ooae135 (2024). 39530053 10.1093/jamiaopen/ooae135 PMC11552621

[251] Hadeed, N. et al. Taming the in-basket-how two simple tools reduced portal message volume in an academic internal medicine clinic. *J .Gen. Intern. Med*. 10.1007/s11606-025-09478-7. (2025). 10.1007/s11606-025-09478-7 PMC12686222 40234358

[252] Lee, J. L. et al. Insecure messaging: how clinicians approach potentially problematic messages from patients. *JAMIA Open* **3**, 576–582, 10.1093/jamiaopen/ooaa051 (2020). 33758796 10.1093/jamiaopen/ooaa051 PMC7969962

[253] Lieu, T. A. et al. Evaluation of attention switching and duration of electronic inbox work among primary care physicians. *JAMA Netw. Open* **4**, e2031856, 10.1001/jamanetworkopen.2020.31856 (2021). 33475754 10.1001/jamanetworkopen.2020.31856 PMC7821028

[254] Akbar, F. et al. Physician stress during electronic health record inbox work: in situ measurement with wearable sensors. *JMIR Med. Inf.* **9**, e24014, 10.2196/24014 (2021). 10.2196/24014 PMC8116996 33908888

[255] Murphy, D. R. et al. An exploration of barriers, facilitators, and suggestions for improving electronic health record inbox-related usability: a qualitative analysis. *JAMA Netw. Open* **2**, e1912638, 10.1001/jamanetworkopen.2019.12638 (2019). 31584683 10.1001/jamanetworkopen.2019.12638 PMC6784746

[256] Hoerter, J.E., Debbaneh, P.M. & Jiang, N. Differences in patient secure message volume among otolaryngologists: a retrospective cohort study. *Ann. Otol. Rhinol. Laryngol.* 34894241264114. 10.1177/00034894241264114. (2024). 10.1177/00034894241264114 39054802

[257] Mandal, S. et al. Quantifying the impact of telemedicine and patient medical advice request messages on physicians’ work-outside-work. *Npj Digit. Med.* **7**. 10.1038/s41746-024-01001-2. (2024). 10.1038/s41746-024-01001-2 PMC10867011 38355913

[258] McAlearney, A. S. et al. Empowering patients during hospitalization: perspectives on inpatient portal use. *Appl. Clin. Inf.* **10**, 103–112, 10.1055/s-0039-1677722 (2019). 10.1055/s-0039-1677722 PMC6374145 30759491

[259] Halterman, A.W. et al. Pediatric resident use, perceptions, and desires for improvement of a clinical secure messaging application. *Comput. Methods Programs Biomed. Update.* **6**. 10.1016/j.cmpbup.2024.100162. (2024).

[260] Lanham, H. J., Leykum, L. K. & Pugh, J. A. Examining the complexity of patient-outpatient care team secure message communication: qualitative analysis. *J. Med. Internet Res.* **20**, e218, 10.2196/jmir.9269 (2018). 29997107 10.2196/jmir.9269 PMC6060302

[261] Wooldridge, A.R. et al. Technology-mediated communication between patients and primary care clinicians and staff: ambiguity in secure messaging. 556–560. 10.1177/1541931213601128. (2016).

[262] Shimada, S. L. et al. Impact of patient-clinical team secure messaging on communication patterns and patient experience: randomized encouragement design trial. *J. Med. Internet Res.* **22**, e22307, 10.2196/22307 (2020). 33206052 10.2196/22307 PMC7710447

[263] Lee, J. L. et al. Too many don’ts and not enough do’s? A Survey of hospitals about their portal instructions for patients. *J. Gen. Intern. Med.* **35**, 1029–1034, 10.1007/s11606-019-05528-z (2020). 31720967 10.1007/s11606-019-05528-z PMC7174450

[264] Rule, A. et al. Primary care staff members’ experiences with managing electronic health record inbox messages. *J. Am. Med. Inf. Assoc.* **32**, 1040–1049, 10.1093/jamia/ocaf067 (2025). 10.1093/jamia/ocaf067 PMC12089763 40298903

[265] Zallman, L. et al. Do medical scribes help primary care providers respond more quickly to out-of-visit tasks?. *J. Am. Board Fam. Med.* **34**, 70–77, 10.3122/jabfm.2021.01.200330 (2021). 33452084 10.3122/jabfm.2021.01.200330

[266] Miller, H. N. et al. Describing current use, barriers, and facilitators of patient portal messaging for research recruitment: Perspectives from study teams and patients at one institution. *J. Clin. Transl. Sci.* **7**, e96, 10.1017/cts.2023.522 (2023). 37125060 10.1017/cts.2023.522 PMC10130833

[267] Hefner, J. L., Sieck, C. J. & Walker, D. M. Patient and physician perspectives on training to improve communication through secure messaging: clarifying the rules of engagement. *Health Care Manag. Rev.* **47**, 3–11, 10.1097/HMR.0000000000000279 (2022). 10.1097/HMR.0000000000000279 32379081

[268] Hefner, J., Sieck, C. & McAlearney, A. Training to optimize collaborative use of an inpatient portal. *Appl. Clin. Inf.* **09**, 558–564, 10.1055/s-0038-1666993 (2018). 10.1055/s-0038-1666993 PMC6059853 30045386

[269] Hyslop, A., Swazo, R. & Smith, J. P. “A friendly reminder” – Improving workflow and efficiency in a pulmonary fellows’ outpatient continuity clinic. *Heart Lung* **63**, 167–174, 10.1016/j.hrtlng.2023.10.007 (2024). 37925749 10.1016/j.hrtlng.2023.10.007

[270] Smith, R. C. et al. Ways to improve workflow and morale in an ophthalmology clinic: survey advice from clinic staff. *J. Biotechnol. Biomed.* **6**, 460–467, 10.26502/jbb.2642-91280108 (2023). 38817776 10.26502/jbb.2642-91280108 PMC11138118

[271] Morrow, D. et al. Contextualizing numeric clinical test results for gist comprehension: implications for EHR patient portals. *J. Exp. Psychol. Appl.* **25**, 41–61, 10.1037/xap0000203 (2019). 30688498 10.1037/xap0000203

[272] Kapoor, A. et al. Comparing the efficacy of targeted and blast portal messaging in message opening rate and anticoagulation initiation in patients with atrial fibrillation in the preventing preventable strokes study II: prospective cohort study. *JMIR Cardio.* **8**, e49590, 10.2196/49590 (2024). 38265849 10.2196/49590 PMC10851125

[273] Taylor Pearson, K. E. Pediatric clinical staff perspectives on secure messaging. *J. Nurs. Care Qual.* **39**, 317–323, 10.1097/NCQ.0000000000000775 (2024). 39172531 10.1097/NCQ.0000000000000775

[274] Alpert, J. M. et al. Integrating patient-centeredness into online patient-clinician communication: a qualitative analysis of clinicians’ secure messaging usage. *Support Care Cancer* **30**, 9851–9857, 10.1007/s00520-022-07408-5 (2022). 36260178 10.1007/s00520-022-07408-5 PMC9580446

[275] Roscoe, R.D. et al. Automated strategy feedback can improve the readability of physicians’ electronic communications to simulated patients. *Int. J. Hum. Comput. Stud.* 176. 10.1016/j.ijhcs.2023.103059. (2023). 10.1016/j.ijhcs.2023.103059 PMC10174593 37193118

[276] Padman, R. et al. eVisit: a pilot study of a new kind of healthcare delivery. *Stud. Health Technol. Inf.* **160**, 262–266 (2010). 20841690

[277] Parpia, C. et al. Evaluation of a secure messaging system in the care of children with medical complexity: mixed methods study. *JMIR Form. Res.* **7**, e42881, 10.2196/42881 (2023). 36821356 10.2196/42881 PMC9999262

[278] Rohrer, J. E. et al. Timely response to secure messages from primary care patients. *Qual. Manag Health Care* **22**, 161–166, 10.1097/QMH.0b013e31828be314 (2013). 23542371 10.1097/QMH.0b013e31828be314

[279] Dyrbye, L. N. et al. Relationships between EHR-based audit log data and physician burnout and clinical practice process measures. *Mayo Clin. Proc.* **98**, 398–409, 10.1016/j.mayocp.2022.10.027 (2023). 36868747 10.1016/j.mayocp.2022.10.027

[280] Jahn, M. A. et al. Usability assessment of secure messaging for clinical document sharing between health care providers and patients. *Appl. Clin. Inf.* **9**, 467–477, 10.1055/s-0038-1660521 (2018). 10.1055/s-0038-1660521 PMC6021963 29949815

[281] Chen, M.L. et al. Analysing common topics of secure patient messages in hidradenitis suppurativa: a text-embedding and natural language-processing approach. Br J Dermatol 2024. 10.1093/bjd/ljae222. 10.1093/bjd/ljae222 38975641

[282] Morrow, D. et al. A multidisciplinary approach to designing and evaluating Electronic Medical Record portal messages that support patient self-care. *J. Biomed. Inf.* **69**, 63–74, 10.1016/j.jbi.2017.03.015 (2017). 10.1016/j.jbi.2017.03.015 PMC5492515 28347856

[283] Garvin, L. A. & Simon, S. R. Prioritizing measures of digital patient engagement: a delphi expert panel study. *J. Med. Internet Res.* **19**, e182, 10.2196/jmir.4778 (2017). 28550008 10.2196/jmir.4778 PMC5466699

[284] Alpert, J. M. et al. Evaluating the SEND eHealth application to improve patients’ secure message writing. *J. Cancer Educ.*10.1007/s13187-024-02491-0 (2024). 39222291 10.1007/s13187-024-02491-0 PMC11978710

[285] Mirsky, J. B. et al. Readability assessment of patient-provider electronic messages in a primary care setting. *J. Am. Med. Inf. Assoc.* **23**, 202–206, 10.1093/jamia/ocv087 (2016). 10.1093/jamia/ocv087 PMC7814916 26177659

[286] Crossley, S. A. et al. Predicting the readability of physicians’ secure messages to improve health communication using novel linguistic features: findings from the ECLIPPSE study. *J. Commun. Heal* **13**, 1–13, 10.1080/17538068.2020.1822726 (2020). 10.1080/17538068.2020.1822726 PMC8300594 34306181

[287] Crossley, S. A. et al. Developing and testing automatic models of patient communicative health literacy using linguistic features: findings from the ECLIPPSE study. *Health Commun.* **36**, 1018–1028, 10.1080/10410236.2020.1731781 (2021). 32114833 10.1080/10410236.2020.1731781 PMC7483831

[288] Schillinger, D. et al. Employing computational linguistics techniques to identify limited patient health literacy: findings from the ECLIPPSE study. *Health Serv. Res.* **56**, 132–144, 10.1111/1475-6773.13560 (2021). 32966630 10.1111/1475-6773.13560 PMC7839650

[289] Schillinger, D. et al. Validity of a computational linguistics-derived automated health literacy measure across race/ethnicity: findings from the ECLIPPSE project. *J. Health Care Poor Underserved* **32**, 347–365, 10.1353/hpu.2021.0067 (2021). 36101652 10.1353/hpu.2021.0067 PMC9467454

[290] Gao, M. et al. Development of a flexible chain of thought framework for automated routing of patient portal messages. *AMIA Annu Symp. Proc.* **2024**, 443–452 (2024). 40417581 PMC12099328

[291] Mao, C. M. & Hovick, S. R. Adding affordances and communication efficacy to the technology acceptance model to study the messaging features of online patient portals among young adults. *Health Commun.* **37**, 307–315, 10.1080/10410236.2020.1838106 (2022). 33243017 10.1080/10410236.2020.1838106

[292] Martin, D. et al. Systematic identification of caregivers of patients living with dementia in the electronic health record: known contacts and natural language processing cohort study. *J. Med. Internet Res.* **27**, e63654, 10.2196/63654 (2025). 40324164 10.2196/63654 PMC12089870

[293] Anderson, B.J. et al. Development and evaluation of a model to manage patient portal messages. NEJM AI 2. 10.1056/AIoa2400354. (2025).

[294] Huang, M. et al. Identification of transportation barriers in patient portal messages via deep semantic embeddings and clustering. *Stud. Health Technol. Inf.* **290**, 794–798, 10.3233/SHTI220188 (2022). 10.3233/SHTI220188 35673127

[295] Duvall, M. J. et al. Portal message language use prior to suicide, suicide attempts, and hospitalization for depression. *Telemed. J. E Health* **28**, 1143–1150, 10.1089/tmj.2021.0318 (2022). 34936819 10.1089/tmj.2021.0318

[296] Duvall, M. et al. Patient portal message characteristics and reported thoughts of self-harm and suicide: a retrospective cohort study. *J. Telemed. Telecare* **27**, 501–508, 10.1177/1357633X19887262 (2021). 31726902 10.1177/1357633X19887262

[297] Tafti, A.P. et al. Artificial intelligence to organize patient portal messages: a journey from an ensemble deep learning text classification to rule-based named entity recognition. 1380–1387. 10.1109/BIBM47256.2019.8982942. (2019).

[298] Si, S. et al. Students need more attention: BERT-based attention model for small data with application to automatic patient message triage. *Proc. Mach. Learn. Res.* **126**, 436–456 (2020).

[299] Yang, J. et al. Development and evaluation of an artificial intelligence-based workflow for the prioritization of patient portal messages. *JAMIA Open* **7**, ooae078, 10.1093/jamiaopen/ooae078 (2024). 39156046 10.1093/jamiaopen/ooae078 PMC11328532

[300] Moon, S. et al. Automated identification of patients’ unmet social needs in clinical text using natural language processing. *Mayo Clin. Proc. Digit. Health* **2**, 411–420, 10.1016/j.mcpdig.2024.06.008 (2024). 39324128 10.1016/j.mcpdig.2024.06.008 PMC11423779

[301] Ren, Y. et al. Automatic uncovering of patient primary concerns in portal messages using a fusion framework of pretrained language models. *J. Am. Med. Inf. Assoc.* **31**, 1714–1724, 10.1093/jamia/ocae144 (2024). 10.1093/jamia/ocae144 PMC11258404 38934289

[302] Baek, J. et al. Assessing patient needs during natural disasters: mixed methods analysis of portal messages sent during Hurricane Harvey. *J. Med. Internet Res.* **23**, e31264, 10.2196/31264 (2021). 34468328 10.2196/31264 PMC8444041

[303] Alpert, J. M. et al. Secure messaging and COVID-19: a content analysis of patient-clinician communication during the pandemic. *Telemed. J. E Health* **28**, 1028–1034, 10.1089/tmj.2021.0316 (2022). 34767741 10.1089/tmj.2021.0316 PMC9293676

[304] Bhandarkar, A. R. et al. Building a natural language processing artificial intelligence to predict suicide-related events based on patient portal message data. *Mayo Clin. Proc. Digit. Health* **1**, 510–518, 10.1016/j.mcpdig.2023.09.001 (2023). 40206315 10.1016/j.mcpdig.2023.09.001 PMC11975696

[305] Sulieman, L. et al. Classifying patient portal messages using convolutional neural networks. *J. Biomed. Inf.* **74**, 59–70, 10.1016/j.jbi.2017.08.014 (2017). 10.1016/j.jbi.2017.08.014 28864104

[306] De, A. et al. Analyzing patient secure messages using a fast health care interoperability resources (FIHR)-based data model: development and topic modeling study. *J. Med. Internet Res.* **23**, e26770, 10.2196/26770 (2021). 34328444 10.2196/26770 PMC8367168

[307] Kiani, H. et al. Improving emergency department visit risk prediction: exploring the operational utility of applied patient portal messages. *AMIA Annu. Symp. Proc.* **2024**, 610–619 (2024). 40417468 PMC12099376

[308] Sulieman, L., Yin, Z. & Malin, B. A. Why patient portal messages indicate risk of readmission for patients with ischemic heart disease. *AMIA Annu. Symp. Proc.* **2019**, 828–837 (2019). 32308879 PMC7153079

[309] Song, Q. et al. Optimizing word embeddings for small dataset: a case study on patient portal messages from breast cancer patients. *Sci. Rep.* **14**, 16117. 10.1038/s41598-024-66319-z (2024). 38997332 10.1038/s41598-024-66319-z PMC11245534

[310] Wang, N. et al. Taxonomy-based prompt engineering to generate synthetic drug-related patient portal messages. *J. Biomed. Inform.* **160**. 10.1016/j.jbi.2024.104752. (2024). 10.1016/j.jbi.2024.104752 39603549

[311] Liu, S. et al. Detecting emergencies in patient portal messages using large language models and knowledge graph-based retrieval-augmented generation. *J. Am. Med. Inf. Assoc.* **32**, 1032–1039, 10.1093/jamia/ocaf059 (2025). 10.1093/jamia/ocaf059 PMC12089757 40220286

[312] Kim, J. et al. Artificial intelligence tools in supporting healthcare professionals for tailored patient care. *NPJ Digit. Med.* **8**, 210, 10.1038/s41746-025-01604-3 (2025). 40240489 10.1038/s41746-025-01604-3 PMC12003912

[313] Chekuri, A. et al. Towards optimizing LLM use in healthcare: identifying patient questions in MyChart messages. *AMIA Annu. Symp. Proc.* **2024**, 232–241 (2024). 40417557 PMC12099336

[314] Alsumait, A. et al. Triage of patient messages sent to the eye clinic via the electronic medical record: a comparative study on AI and human triage performance. *J. Clin. Med.* **14**. 10.3390/jcm14072395. (2025). 10.3390/jcm14072395 PMC11989310 40217845

[315] Pham, J. H. et al. Large language model triaging of simulated nephrology patient inbox messages. *Front. Artif. Intell.* **7**, 1452469, 10.3389/frai.2024.1452469 (2024). 39315245 10.3389/frai.2024.1452469 PMC11417033

[316] Garcia, P. et al. Artificial intelligence-generated draft replies to patient inbox messages. *JAMA Netw. Open* **7**, e243201, 10.1001/jamanetworkopen.2024.3201 (2024). 38506805 10.1001/jamanetworkopen.2024.3201 PMC10955355

[317] Small, W. R. et al. Large language model–based responses to patients’ in-basket messages. *JAMA Netw. Open* **7**, e2422399, 10.1001/jamanetworkopen.2024.22399 (2024). 39012633 10.1001/jamanetworkopen.2024.22399 PMC11252893

[318] Tailor, P. D. et al. A comparative study of responses to retina questions from either experts, expert-edited large language models, or expert-edited large language models alone. *Ophthalmol. Sci.* **4**, 100485, 10.1016/j.xops.2024.100485 (2024). 38660460 10.1016/j.xops.2024.100485 PMC11041826

[319] Tailor, P. D. et al. Appropriateness of ophthalmology recommendations from an online chat-based artificial intelligence model. *Mayo Clin. Proc. Digit Health* **2**, 119–128, 10.1016/j.mcpdig.2024.01.003 (2024). 38577703 10.1016/j.mcpdig.2024.01.003 PMC10994056

[320] Proctor, S., Lawton, G. & Sinha, S. An AI-powered strategy for managing patient messaging load and reducing burnout. *Appl. Clin. Inf.* **16**, 747–752, 10.1055/a-2576-0579 (2025). 10.1055/a-2576-0579 PMC12328029 40199518

[321] Chen, S. et al. The effect of using a large language model to respond to patient messages. *Lancet Digit Health* **6**, e379–e381, 10.1016/S2589-7500(24)00060-8 (2024). 38664108 10.1016/S2589-7500(24)00060-8 PMC11829255

[322] Liu, S. et al. Leveraging large language models for generating responses to patient messages—a subjective analysis. *J. Am. Med. Inf. Assoc.* **31**, 1367–1379, 10.1093/jamia/ocae052 (2024). 10.1093/jamia/ocae052 PMC11105129 38497958

[323] Cavalier, J. S. et al. Ethics in patient preferences for artificial intelligence–drafted responses to electronic messages. *JAMA Netw. Open* **8**, e250449, 10.1001/jamanetworkopen.2025.0449 (2025). 40067301 10.1001/jamanetworkopen.2025.0449 PMC11897835

[324] Athavale, A. et al. The potential of chatbots in chronic venous disease patient management. *JVS Vasc. Insights* **1**. 10.1016/j.jvsvi.2023.100019. (2023). 10.1016/j.jvsvi.2023.100019 PMC10497234 37701430

[325] Nov, O., Singh, N. & Mann, D. Putting ChatGPT’s medical advice to the (turing) test: survey study. *JMIR Med. Educ.* **9**, e46939, 10.2196/46939 (2023). 37428540 10.2196/46939 PMC10366957

[326] English, E. et al. Utility of artificial intelligence-generative draft replies to patient messages. *JAMA Netw. Open* **7**, e2438573, 10.1001/jamanetworkopen.2024.38573 (2024). 39401041 10.1001/jamanetworkopen.2024.38573 PMC11581472

[327] Kim, J. et al. Patient perspectives on large language model responses to patient messages. 10.2139/ssrn.4867523. .(2024)

[328] Robinson, E. J. et al. Physician vs. AI-generated messages in urology: evaluation of accuracy, completeness, and preference by patients and physicians. *World J. Urol.* **43**, 48, 10.1007/s00345-024-05399-y (2024). 39729119 10.1007/s00345-024-05399-y PMC11680670

[329] Scott, M. et al. Assessing artificial intelligence–generated responses to urology patient in-basket messages. *Urol. Pract.* **11**, 793–798, 10.1097/UPJ.0000000000000637 (2024). 39162591 10.1097/UPJ.0000000000000637

[330] Soroudi, D. et al. Comparing provider and ChatGPT responses to breast reconstruction patient questions in the electronic health record. *Ann. Plast. Surg.* **93**, 541–545, 10.1097/SAP.0000000000004090 (2024). 39445873 10.1097/SAP.0000000000004090

[331] Hao, Y. et al. Retrospective comparative analysis of prostate cancer in-basket messages: responses from closed-domain large language models versus clinical teams. *Mayo Clin. Proc. Digit. Health* **3**. 10.1016/j.mcpdig.2025.100198. (2025). 10.1016/j.mcpdig.2025.100198 PMC11932704 40130001

[332] Hong, C. et al. Application of unified health large language model evaluation framework to in-basket message replies: bridging qualitative and quantitative assessments. *J. Am. Med. Inf. Assoc*. 10.1093/jamia/ocaf023. (2025). 10.1093/jamia/ocaf023 PMC12005619 40063081

[333] Kaur, A. et al. Primary care providers acceptance of generative ai responses to patient portal messages. *Appl. Clin. Inform.*10.1055/a-2565-9155. (2025). 10.1055/a-2565-9155 PMC12310298 40132987

[334] Tse, G. et al. Large language model responses to adolescent patient and proxy messages. *JAMA Pediatr.* **179**, 93–94, 10.1001/jamapediatrics.2024.4438 (2025). 39495530 10.1001/jamapediatrics.2024.4438 PMC11536304

[335] Bootsma-Robroeks CMHHT, Workum, J. D. et al. AI-generated draft replies to patient messages: exploring effects of implementation. *Front. Digit. Health* **7**, 1588143, 10.3389/fdgth.2025.1588143 (2025). 40575383 10.3389/fdgth.2025.1588143 PMC12198195

[336] Andreadis, K. et al. Bridging gaps with generative AI: enhancing hypertension monitoring through patient and provider insights. *Stud. Health Technol. Inf.* **316**, 939–943, 10.3233/SHTI240565 (2024). 10.3233/SHTI240565 39176946

[337] Reynolds, K. et al. Comparing the quality of ChatGPT- and physician-generated responses to patients’ dermatology questions in the electronic medical record. *Clin. Exp. Dermatol.* **49**, 715–718, 10.1093/ced/llad456 (2024). 38180108 10.1093/ced/llad456

[338] Kim, J. et al. Perspectives on artificial intelligence-generated responses to patient messages. *JAMA Netw. Open* **7**, e2438535, 10.1001/jamanetworkopen.2024.38535 (2024). 39412810 10.1001/jamanetworkopen.2024.38535 PMC11581642

[339] Luo, M. et al. Assessing empathy in large language models with real-world physician-patient interactions. 6510–6519. 10.1109/BigData62323.2024.10825307. (2024).

[340] Liu, S. et al. Using large language model to guide patients to create efficient and comprehensive clinical care message. *J. Am. Med. Inf. Assoc.* **31**, 1665–1670, 10.1093/jamia/ocae142 (2024). 10.1093/jamia/ocae142 PMC11258400 38917441

[341] Yan, S. et al. Prompt engineering on leveraging large language models in generating response to InBasket messages. *J. Am. Med. Inf. Assoc.* **31**, 2263–2270, 10.1093/jamia/ocae172 (2024). 10.1093/jamia/ocae172 PMC11413421 39028970

[342] Kaur, A. et al. Automating responses to patient portal messages using generative AI. 10.1101/2024.04.25.24306183. 10.1055/a-2565-9155 PMC12310298 40132987

[343] Lee, N. S. et al. Use of a medical communication framework to assess the quality of generative artificial intelligence replies to primary care patient portal messages: content analysis. *JMIR Form. Res.* **9**, e71966, 10.2196/71966 (2025). 40743559 10.2196/71966 PMC12313158

[344] Biro, J. M. et al. Opportunities and risks of artificial intelligence in patient portal messaging in primary care. *NPJ Digit. Med.* **8**, 222, 10.1038/s41746-025-01586-2 (2025). 40275104 10.1038/s41746-025-01586-2 PMC12022076

[345] Foresman, G. et al. Patient perspectives on artificial intelligence in health care: focus group study for diagnostic communication and tool implementation. *J. Particip. Med.* **17**, e69564, 10.2196/69564 (2025). 40705399 10.2196/69564 PMC12288699

[346] Green, A. R. et al. Characterizing patient portal use of people with cognitive impairment and potentially inappropriate medications. *J. Am. Geriatr. Soc.* **73**, 750–758, 10.1111/jgs.19284 (2025). 39578983 10.1111/jgs.19284 PMC11908959

[347] Lieu, T. A. et al. Pharmacist vs physician management of e-visit requests for COVID-19 medication: a randomized clinical trial. *J. Manag Care Spec. Pharm.* **31**, 189–197, 10.18553/jmcp.2025.31.2.189 (2025). 39912817 10.18553/jmcp.2025.31.2.189 PMC11801361

[348] Shimada, S. L. et al. An analysis of patient-provider secure messaging at two Veterans Health Administration medical centers: message content and resolution through secure messaging. *J. Am. Med. Inf. Assoc.* **24**, 942–949, 10.1093/jamia/ocx021 (2017). 10.1093/jamia/ocx021 PMC7651909 28371896

[349] Heisey-Grove, D. et al. Classification of patient- and clinician-generated secure messages using a theory-based taxonomy. *Health Sci. Rep.* **4**, e295, 10.1002/hsr2.295 (2021). 34084944 10.1002/hsr2.295 PMC8142627

[350] Flickinger, T. E. et al. Communication between patients, peers, and care providers through a mobile health intervention supporting medication-assisted treatment for opioid use disorder. *Patient Educ. Couns.* **105**, 2110–2115, 10.1016/j.pec.2022.02.014 (2022). 35260260 10.1016/j.pec.2022.02.014 PMC10112280

[351] Davoudi, A. et al. Identifying medication-related intents from a bidirectional text messaging platform for hypertension management using an unsupervised learning approach: retrospective observational pilot study. *J. Med. Internet Res.* **24**, e36151, 10.2196/36151 (2022). 35767327 10.2196/36151 PMC9280462

[352] Robinson, S. A. et al. Secure messaging for diabetes management: content analysis. *JMIR Diabetes* **8**, e40272, 10.2196/40272 (2023). 36951903 10.2196/40272 PMC10131591

[353] Green, A. R. et al. Use of the patient portal to discuss medications among people with dementia and their care partners: patient portal correspondence about medications. *J. Gen. Intern. Med.* **39**, 3164–3171, 10.1007/s11606-024-09064-3 (2024). 39354256 10.1007/s11606-024-09064-3 PMC11618272

[354] Anderson, B. et al. Empowering care teams: redefining message management to enhance care delivery and alleviate oncologist burnout. *JNCCN J. Natl. Compr. Cancer Netw.* **22**, 664–669, 10.6004/jnccn.2024.7055 (2024). 10.6004/jnccn.2024.7055 39602886

[355] Ramirez-Zohfeld, V. et al. Use of electronic health records by older adults, 85 Years and older, and their caregivers. *J. Am. Geriatr. Soc.* **68**, 1078–1082, 10.1111/jgs.16393 (2020). 32159860 10.1111/jgs.16393

[356] Sieck, C. J. et al. Understanding secure messaging in the inpatient environment: a new avenue for communication and patient engagement. *Appl. Clin. Inf.* **9**, 860–868, 10.1055/s-0038-1675814 (2018). 10.1055/s-0038-1675814 PMC6281442 30517969

[357] Yin, Z. et al. The therapy is making me sick: how online portal communications between breast cancer patients and physicians indicate medication discontinuation. *J. Am. Med. Inf. Assoc.* **25**, 1444–1451, 10.1093/jamia/ocy118 (2018). 10.1093/jamia/ocy118 PMC7646923 30380083

[358] Carter, R. R. et al. Assessing mental models from communications: patient, family, and care team messaging within the Hospital. *Proc. Hum. Factors Erg. Soc. Annu. Meet.* **63**, 653–657, 10.1177/1071181319631440 (2019).

[359] Meier-Diedrich, E. et al. Patient-health care professional communication via a secure web-based portal in severe mental health conditions: qualitative analysis of secure messages. *JMIR Form. Res.* **9**, e63713, 10.2196/63713 (2025). 40577718 10.2196/63713 PMC12254702

[360] Yin, Z. et al. Patient messaging content associated with initiating hormonal therapy after a breast cancer diagnosis. *AMIA Annu. Symp. Proc.* **2019**, 962–971 (2019). 32308893 PMC7153093

[361] Heisey-Grove, D. M. & DeShazo, J. P. Look who’s talking: application of a theory-based taxonomy to patient-clinician E-mail messages. *Telemed. J. E Health* **26**, 1345–1352, 10.1089/tmj.2019.0192 (2020). 32074474 10.1089/tmj.2019.0192

[362] Shetty, V.A. et al. Discussions of cannabis over patient portal secure messaging: content analysis. *J. Med. Internet Res.* **26**. 10.2196/63311. (2024). 10.2196/63311 PMC11671783 39666375

[363] Palmer, S.K. et al. Deep inferior epigastric perforator in-basket? evaluation of patient electronic communication following autologous breast reconstruction. *Aesthet. Surg. J*. 10.1093/asj/sjaf177. (2025). 10.1093/asj/sjaf177 40922661

[364] Hogan, T. P. et al. Patient centeredness in electronic communication: evaluation of patient-to-health care team secure messaging. *J. Med. Internet Res.* **20**, e82, 10.2196/jmir.8801 (2018). 29519774 10.2196/jmir.8801 PMC5864998

[365] Spencer, C., Loehr, K. & Byrd, A. Patient and family perpetrated cyber-incivility and cyber-aggression within healthcare: a cross-sectional descriptive study. *SAGE Open Nurs.* **9**, 23779608231158970, 10.1177/23779608231158970 (2023). 36923238 10.1177/23779608231158970 PMC10009025

[366] Alpert, J. M., Dyer, K. E. & Lafata, J. E. Patient-centered communication in digital medical encounters. *Patient Educ. Couns.* **100**, 1852–1858, 10.1016/j.pec.2017.04.019 (2017). 28522229 10.1016/j.pec.2017.04.019 PMC5573682

[367] Robinson, J. R. et al. Complexity of medical decision-making in care provided by surgeons through patient portals. *J. Surg. Res.* **214**, 93–101, 10.1016/j.jss.2017.02.077 (2017). 28624066 10.1016/j.jss.2017.02.077 PMC5474935

[368] Kim, J. et al. Patient-centered research through artificial intelligence to identify priorities in cancer care. *JAMA Oncol.* **11**, 630–635, 10.1001/jamaoncol.2025.0694 (2025). 40272833 10.1001/jamaoncol.2025.0694 PMC12022861

[369] ONC’s Cures Act Final Rule | HealthIT.gov n.d. https://www.healthit.gov/topic/oncs-cures-act-final-rule (accessed November 6, 2024).

[370] Xie, J. et al. Ensuring adolescent patient portal confidentiality in the age of the cures act final rule. *J. Adolesc. Health* **69**, 933–939, 10.1016/j.jadohealth.2021.09.009 (2021). 34666956 10.1016/j.jadohealth.2021.09.009

[371] Ip, W. et al. Assessment of prevalence of adolescent patient portal account access by guardians. *JAMA Netw. Open* **4**, e2124733, 10.1001/jamanetworkopen.2021.24733 (2021). 34529064 10.1001/jamanetworkopen.2021.24733 PMC8446820

[372] Sisk, B. A. et al. Acceptability of adolescent portal access policies to parents and adolescents: a delphi study. *J. Adolesc. Health* **76**, 448–454, 10.1016/j.jadohealth.2024.10.021 (2025). 39614854 10.1016/j.jadohealth.2024.10.021

[373] Gleason, K. T. et al. Use of the patient portal among older adults with diagnosed dementia and their care partners. *Alzheimers Dement*10.1002/alz.13354 (2023). 37354066 10.1002/alz.13354 PMC10808947

[374] Semere, W. et al. Care partner engagement in secure messaging between patients with diabetes and their clinicians: cohort study. *JMIR Diab.* **9**, e49491, 10.2196/49491 (2024). 10.2196/49491 PMC10891488 38335020

[375] Pecina, J, Duvall, M.J. & North, F. Frequency of and factors associated with care partner proxy interaction with health care teams using patient portal accounts. *Telemed. E Health* 26, 1368–1372. 10.1089/tmj.2019.0208. (2020). 10.1089/tmj.2019.0208 31971889

[376] Benda, N. C. et al. Identifying nonpatient authors of patient portal secure messages in oncology: a proof-of-concept demonstration of natural language processing methods. *JCO Clin. Cancer Inf.* **6**, e2200071, 10.1200/CCI.22.00071 (2022). 10.1200/CCI.22.00071 PMC10476725 36542818

[377] Balyan, R. et al. Using natural language processing and machine learning to classify health literacy from secure messages: the ECLIPPSE study. *PLoS ONE* **14**, e0212488, 10.1371/journal.pone.0212488 (2019). 30794616 10.1371/journal.pone.0212488 PMC6386302

[378] Holmgren, A. J. et al. Association between billing patient portal messages as e-visits and patient messaging volume. *JAMA* **329**, 339, 10.1001/jama.2022.24710 (2023). 36607621 10.1001/jama.2022.24710 PMC10408262

[379] Alpert, J. M. et al. Qualitative analysis of patients’ and physicians’ attitudes and behaviors toward billing patient portal messages. *Ann. Intern. Med.* **177**, 1734–1736, 10.7326/ANNALS-24-00560 (2024). 39401435 10.7326/ANNALS-24-00560

[380] Dunlay, S. M. et al. Implementation of billing for patient portal messages as E-visits in a large integrated health system. *Ann. Intern Med.* **178**, 11–19, 10.7326/ANNALS-24-01711 (2025). 39745809 10.7326/ANNALS-24-01711

[381] Ko, DG, Tachinardi, U & Warm, EJ. Secure messaging telehealth billing in the digital age: moving beyond time-based metrics. *J. Am. Med. Inf. Assoc*. 10.1093/jamia/ocae250. (2024). 10.1093/jamia/ocae250 PMC11648735 39325492

[382] Oke, I. et al. Trends in billing secure messages at ophthalmology practices across the United States. *Ophthalmol. Sci.* **5**. 10.1016/j.xops.2024.100683. (2025). 10.1016/j.xops.2024.100683 PMC11925567 40114713

[383] Reynolds, TL, Ali, N & Zheng, K. What do patients and caregivers want? A systematic review of user suggestions to improve patient portals n.d. PMC8075519 33936483

[384] Raisa, A. et al. Identifying the mechanisms of patient-centred communication in secure messages between clinicians and cancer patients. *PEC Innov.* **2**, 100161, 10.1016/j.pecinn.2023.100161 (2023). 37384151 10.1016/j.pecinn.2023.100161 PMC10294087

[385] Mccormack, J. et al. Direct secure messaging in practice: addressing workflow challenges…19th World Congress on medical and health informatics, July 8-12, 2023, New South Wales, Australia. *Stud. Health Technol. Inf.* **310**, 189–193, 10.3233/SHTI230953 (2023). 10.3233/SHTI230953 38269791

[386] Rodriguez DV, et al. Development of a GenAI-powered hypertension management assistant: early development phases and architectural design. 350–359. 10.1109/ICHI61247.2024.00052. (2024).

[387] Ball, S. L. et al. Clinician and staff experiences with frustrated patients during an electronic health record transition: a qualitative case study. *BMC Health Serv. Res.* **24**, 535. 10.1186/s12913-024-10974-5 (2024). 38671473 10.1186/s12913-024-10974-5 PMC11046755

[388] Alpert, J. M. et al. Twenty-first Century bedside manner: exploring patient-centered communication in secure messaging with cancer patients. *J. Cancer Educ.* **36**, 16–24, 10.1007/s13187-019-01592-5 (2021). 31342283 10.1007/s13187-019-01592-5

[389] Gold, K. J. et al. Patient-reported reasons for sending portal messages: a survey of use in a family medicine department. *J. Gen. Intern Med.* **39**, 2608–2611, 10.1007/s11606-024-08815-6 (2024). 38831243 10.1007/s11606-024-08815-6 PMC11436532

[390] Hu D, et al. When AI writes back: ethical considerations by physicians on AI-drafted patient message replies. 10.48550/ARXIV.2508.13217. (2025). PMC12919555 41726446

[391] Reddy, S. Generative AI in healthcare: an implementation science informed translational path on application, integration and governance. *Implement Sci.* **19**, 27, 10.1186/s13012-024-01357-9 (2024). 38491544 10.1186/s13012-024-01357-9 PMC10941464

[392] Ren, Y. et al. Classification of patient portal messages with BERT-based Language Models. 176–182. 10.1109/ICHI57859.2023.00033. (2023).

[393] Van Buchem, M. M. et al. Applying natural language processing to patient messages to identify depression concerns in cancer patients. *J. Am. Med. Inf. Assoc.* **31**, 2255–2262, 10.1093/jamia/ocae188 (2024). 10.1093/jamia/ocae188 PMC11413442 39018490

[394] Clusmann, J. et al. The future landscape of large language models in medicine. *Commun. Med.* **3**, 141, 10.1038/s43856-023-00370-1 (2023). 37816837 10.1038/s43856-023-00370-1 PMC10564921

[395] Haider, S. A. et al. The development and evaluation of a retrieval-augmented generation large language model virtual assistant for postoperative instructions. *Bioengineering* **12**, 1219, 10.3390/bioengineering12111219 (2025). 41301175 10.3390/bioengineering12111219 PMC12649765

[396] Yim, D. et al. Preliminary evidence of the use of generative AI in health care clinical services: systematic narrative review. *JMIR Med. Inf.* **12**, e52073, 10.2196/52073 (2024). 10.2196/52073 PMC10993141 38506918

