# Supplementary material for: A scoping review of studies on secure messaging through patient portals: persistent challenges and potential solutions
Source: Npj Health Syst. 2026 May 24;3:34. doi: 10.1038/s44401-026-00091-2 (PMC13354250; doi:10.1038/s44401-026-00091-2)
Supplement: Supplementary file 1 — Supplementary information [file 44401_2026_91_MOESM1_ESM.pdf]

Preferred Reporting Items for Systematic reviews and Meta-Analyses extension for Scoping Reviews (PRISMA-ScR) Checklist

SECTION	ITEM	PRISMA-ScR CHECKLIST ITEM	REPORTED ON PAGE #
**TITLE**			
Title	1	Identify the report as a scoping review.	P1
**ABSTRACT**			
Structured summary	2	Provide a structured summary that includes (as applicable): background, objectives, eligibility criteria, sources of evidence, charting methods, results, and conclusions that relate to the review questions and objectives.	P1-23
**INTRODUCTION**			
Rationale	3	Describe the rationale for the review in the context of what is already known. Explain why the review questions/objectives lend themselves to a scoping review approach.	P3-4
Objectives	4	Provide an explicit statement of the questions and objectives being addressed with reference to their key elements (e.g., population or participants, concepts, and context) or other relevant key elements used to conceptualize the review questions and/or objectives.	P4
**METHODS**			
Protocol and registration	5	Indicate whether a review protocol exists; state if and where it can be accessed (e.g., a Web address); and if available, provide registration information, including the registration number.	P21
Eligibility criteria	6	Specify characteristics of the sources of evidence used as eligibility criteria (e.g., years considered, language, and publication status), and provide a rationale.	P20
Information sources*	7	Describe all information sources in the search (e.g., databases with dates of coverage and contact with authors to identify additional sources), as well as the date the most recent search was executed.	P20
Search	8	Present the full electronic search strategy for at least 1 database, including any limits used, such that it could be repeated.	Supplementary Table 1
Selection of sources of evidence†	9	State the process for selecting sources of evidence (i.e., screening and eligibility) included in the scoping review.	P20
Data charting process‡	10	Describe the methods of charting data from the included sources of evidence (e.g., calibrated forms or forms that have been tested by the team before their use, and whether data charting was done independently or in duplicate) and any processes for obtaining and confirming data from investigators.	P21-22
Data items	11	List and define all variables for which data were sought and any assumptions and simplifications made.	Supplementary Table 2
Critical appraisal of individual sources of evidence§	12	If done, provide a rationale for conducting a critical appraisal of included sources of evidence; describe the methods used and how this information was used in any data synthesis (if appropriate).	n/a

SECTION	ITEM	PRISMA-ScR CHECKLIST ITEM	REPORTED ON PAGE #
Synthesis of results	13	Describe the methods of handling and summarizing the data that were charted.	P21-23
**RESULTS**			
Selection of sources of evidence	14	Give numbers of sources of evidence screened, assessed for eligibility, and included in the review, with reasons for exclusions at each stage, ideally using a flow diagram.	P4-5
Characteristics of sources of evidence	15	For each source of evidence, present characteristics for which data were charted and provide the citations.	P7-14
Critical appraisal within sources of evidence	16	If done, present data on critical appraisal of included sources of evidence (see item 12).	n/a
Results of individual sources of evidence	17	For each included source of evidence, present the relevant data that were charted that relate to the review questions and objectives.	P7-14
Synthesis of results	18	Summarize and/or present the charting results as they relate to the review questions and objectives.	P7-14
**DISCUSSION**			
Summary of evidence	19	Summarize the main results (including an overview of concepts, themes, and types of evidence available), link to the review questions and objectives, and consider the relevance to key groups.	P7-14
Limitations	20	Discuss the limitations of the scoping review process.	P19
Conclusions	21	Provide a general interpretation of the results with respect to the review questions and objectives, as well as potential implications and/or next steps.	P19
**FUNDING**			
Funding	22	Describe sources of funding for the included sources of evidence, as well as sources of funding for the scoping review. Describe the role of the funders of the scoping review.	P23

JBI = Joanna Briggs Institute; PRISMA-ScR = Preferred Reporting Items for Systematic reviews and Meta-Analyses extension for Scoping Reviews.
* Where *sources of evidence* (see second footnote) are compiled from, such as bibliographic databases, social media platforms, and Web sites.
† A more inclusive/heterogeneous term used to account for the different types of evidence or data sources (e.g., quantitative and/or qualitative research, expert opinion, and policy documents) that may be eligible in a scoping review as opposed to only studies. This is not to be confused with *information sources* (see first footnote).
‡ The frameworks by Arksey and O'Malley (6) and Levac and colleagues (7) and the JBI guidance (4, 5) refer to the process of data extraction in a scoping review as data charting.
§ The process of systematically examining research evidence to assess its validity, results, and relevance before using it to inform a decision. This term is used for items 12 and 19 instead of "risk of bias" (which is more applicable to systematic reviews of interventions) to include and acknowledge the various sources of evidence that may be used in a scoping review (e.g., quantitative and/or qualitative research, expert opinion, and policy document).

From: Tricco AC, Lillie E, Zarin W, O'Brien KK, Colquhoun H, Levac D, et al. PRISMA Extension for Scoping Reviews (PRISMAScR): Checklist and Explanation. Ann Intern Med. 2018;169:467–473. doi: 10.7326/M18-0850.

Supplementary Table 1. Database Search Queries

Database	Search Query
PubMed	(("secure messag*"[Title/Abstract] OR "portal messag*"[Title/Abstract] OR "inbasket messag*"[Title/Abstract] OR "in-basket messag*"[Title/Abstract] OR "inbox messag*"[Title/Abstract] OR "in-box messag*"[Title/Abstract] OR "mychart messag*"[Title/Abstract] OR "healthelife messag*"[Title/Abstract]) AND patient*[Title/Abstract]) OR "patient-initiated messag*"[Title/Abstract] OR "patient-sent messag*"[Title/Abstract] OR "patient messag*"[Title/Abstract], Filters: from 2009 - 2025
ACM Digital Library	[[[[Abstract: "secure messag*"] OR [Abstract: "portal messag*"] OR [Abstract: "inbasket messag*"] OR [Abstract: "in-basket messag*"] OR [Abstract: "inbox messag*"] OR [Abstract: "in-box messag*"] OR [Abstract: "mychart messag*"] OR [Abstract: "healthelife messag*"]] AND [Abstract: patient*]] OR [All: "patient-initiated messag*"] OR [All: "patient-sent messag*"] OR [All: "patient messag*"]] AND [All:))] AND [E-Publication Date: (01/01/2009 TO 12/31/2025)]
IEEE Xplore	("Abstract":"secure messag*" OR "Abstract":"portal messag*" OR "Abstract":"inbasket messag*" OR "Abstract":"in-basket messag*" OR "Abstract":"inbox messag*" OR "Abstract":"in-box messag*" OR "Abstract":"mychart messag*" OR "Abstract":"healthelife messag*" OR "Abstract":"patient-initiated messag*" OR "Abstract":"patient-sent messag*" OR "Abstract":"patient messag*"<br>) AND "Abstract":patient*<br>Filters Applied: 2009 - 2025
CINAHL	XB (("secure messag*" AND patient*) OR ("portal messag*" AND patient*) OR ("inbasket messag*" AND patient*) OR ("in-basket messag*" AND patient*) OR ("inbox messag*" AND patient*) OR ("in-box messag*" AND patient*) OR ("mychart messag*" AND patient*) OR ("healthelife messag*" AND patient*) OR "patient-initiated messag*" OR "patient-sent messag*" OR "patient messag*")
CENTRAL	(secure NEXT messag* AND patient*) OR (portal NEXT messag* AND patient*) OR (inbasket NEXT messag* AND patient*) OR (in-basket NEXT messag* AND patient*) OR (inbox NEXT messag* AND patient*) OR (in-box NEXT messag* AND patient*) OR (mychart NEXT messag* AND patient*) OR (healthelife NEXT messag* AND patient*) OR patient-initiated NEXT messag* OR patient-sent NEXT messag* OR patient NEXT messag* in Title Abstract Keyword - with Cochrane Library publication date Between Jan 2009 and Dec 2025 (Word variations have been searched)
Scopus	Search with in article Title, Abstract, Keywords, ("secure messag*" AND patient*) OR ("portal messag*" AND patient*) OR ("inbasket messag*" AND patient*) OR ("in-basket messag*" AND patient*) OR ("inbox messag*" AND patient*) OR ("in-box messag*" AND patient*) OR ("mychart messag*" AND patient*) OR ("healthelife messag*" AND patient*) OR "patient-initiated messag*" OR "patient-sent messag*" OR "patient messag*"
Web of Science	AB=(("secure messag*" AND patient*) OR ("portal messag*" AND patient*) OR ("inbasket messag*" AND patient*) OR ("in-basket messag*" AND patient*) OR ("inbox messag*" AND patient*) OR ("in-box messag*" AND patient*) OR ("mychart messag*" AND patient*) OR ("healthelife messag*" AND patient*) OR "patient-initiated messag*" OR "patient-sent messag*" OR "patient messag*")

Supplementary Table 2. Data Extraction Instruments

Domain	Field / Variable	Response options / description
Bibliographic	Study ID	Free text
	Title	Free text
	Publication venue	Free text
	Year of publication	Free text
	Lead author	Free text
	Notes	Free text
Study objectives	Objective category	Multi-select: Message Content Analyses; User Experience and Engagement Assessment; Clinical Outcomes / Efficacy Evaluation; Technology and Application Design; Secure Messaging Adoption / Usage Analysis; Proxy Use and Shared Access; Health / Technology Literacy and Education; Billing and Reimbursement Policy
	Objective (verbatim)	Free text (copied from article)
	Other objective	Free text
Design and methods	Study design / methodology	Multi-select: interview; survey; focus group; narrative/thematic/content analysis; meta-analysis; statistical analysis; machine learning; natural language processing; workflow simulation; randomized controlled trial; retrospective study (case series, chart review, historical cohort); mixed methods; other
	Additional notes on design/method	Free text
Scope and data source	Study scope / data volume	National aggregate; Regional/community aggregate; Clinical cohort; Case study; Other
	Data source	EHR/portal message corpus; survey; interview; other
	Site scope	Single site; Multi-site; Not reported
Population	Population characteristics	Healthcare provider; Infants/children; Adolescents/adults; Elderly; Pregnant women; Military; Veterans; Disabled populations; Racial/ethnic or socioeconomic groups; Not specified; Other
	Notes on population	Free text
Clinical domain	Health condition/domain	Diabetes; Cancer/oncology; Cardiovascular; Other chronic; Mental health; Injuries/trauma; Respiratory; COVID-19; Other infectious; Neurologic; Reproductive; Pediatric; Preventive/wellness; Not specified; Other
	Notes on condition	Free text
Healthcare setting	Service types / facilities	Primary care; Specialist care; Hospital; Outpatient clinic; Nursing home; Home healthcare; Telemedicine; Rehabilitation; Emergency; Palliative/hospice; Not specified; Other
	Notes on setting	Free text
Context tags	Specific event	Free text (e.g., COVID-19, disaster, crisis)
Outcomes and findings	Outcomes measured	Free text
	Results	Free text
	Recommendations / future implementation	Free text
Other	Open-ended notes	Free text
